# Cross-sectoral impacts of the 2018–2019 Central European drought and climate resilience in the German part of the Elbe River basin

**DOI:** 10.1007/s10113-023-02032-3

**Published:** 2023-02-01

**Authors:** Tobias Conradt, Henry Engelhardt, Christoph Menz, Sergio M. Vicente-Serrano, Begoña Alvarez Farizo, Dhais Peña-Angulo, Fernando Domínguez-Castro, Lars Eklundh, Hongxiao Jin, Boris Boincean, Conor Murphy, J. Ignacio López-Moreno

**Affiliations:** 1 Potsdam Institute for Climate Impact Research, Telegrafenberg A31, 14473 Potsdam, Germany; 2 Instituto Pirenaico de Ecología Consejo Superior de Investigaciones Científicas (IPE–CSIC), Zaragoza, Spain; 3 Aragonese Agency for Research and Development Researcher (ARAID), Zaragoza, Spain; 4 Department of Geography, University of Zaragoza, Zaragoza, Spain; 5 Department of Physical Geography and Ecosystem Science, Lund University, Lund, Sweden; 6 Selectia Research Institute of Field Crops, Bălți, Moldova; 7 Irish Climate Analysis and Research UnitS (ICARUS), Department of Geography, Maynooth University, Maynooth, Ireland

**Keywords:** Central European drought, Elbe River basin, Eastern Germany, Drought indices, Drought impacts, Cross-sectoral

## Abstract

The 2018–2019 Central European drought was probably the most extreme in Germany since the early sixteenth century. We assess the multiple consequences of the drought for natural systems, the economy and human health in the German part of the Elbe River basin, an area of 97,175 km^2^ including the cities of Berlin and Hamburg and contributing about 18% to the German GDP. We employ meteorological, hydrological and socio-economic data to build a comprehensive picture of the drought severity, its multiple effects and cross-sectoral consequences in the basin. Time series of different drought indices illustrate the severity of the 2018–2019 drought and how it progressed from meteorological water deficits via soil water depletion towards low groundwater levels and river runoff, and losses in vegetation productivity. The event resulted in severe production losses in agriculture (minus 20–40% for staple crops) and forestry (especially through forced logging of damaged wood: 25.1 million tons in 2018–2020 compared to only 3.4 million tons in 2015–2017), while other economic sectors remained largely unaffected. However, there is no guarantee that this socio-economic stability will be sustained in future drought events; this is discussed in the light of 2022, another dry year holding the potential for a compound crisis. Given the increased probability for more intense and long-lasting droughts in most parts of Europe, this example of actual cross-sectoral drought impacts will be relevant for drought awareness and preparation planning in other regions.

**Supplementary Information:**

The online version contains supplementary material available at 10.1007/s10113-023-02032-3.

## Introduction

The Central European drought of 2018–2019 was a compound event with two consecutive years of exceptionally low precipitation. Through depleted soil water storage the event also extended into 2020. This drought was probably the most severe in Central Europe for centuries, perhaps even for more than two millennia (Büntgen et al. 2021). One has to go back to the first half of the sixteenth century to find heat and drought of comparable magnitudes in this region (Brázdil et al. 2020; Büntgen et al. 2021; Orth et al. 2016; Wetter et al. 2014). In 2022, large parts of Europe experienced yet another exceptional drought, but our analysis focuses on the years 2018–2020 owing to the lagged availability of socio-economic data.

As is typical with such exceptional events, scientific assessments of impacts began to emerge during and soon after the drought. Most frequent among these early responses were studies about crops and forests (e.g. Schuldt et al. 2020; Obladen et al. 2021) or vegetation in general (Bastos et al. 2021), with many assessments involving remote sensing (Reinermann et al. 2019; Buras et al. 2020; Ahmed et al. 2021; Kowalski et al. 2022). A review of media articles researching the drought impact on drinking water supply from private wells in Germany was also conducted (Rickert et al. 2022).

Here we give a broader account of the multisectoral impacts of the 2018–2019 drought in the German part of the Elbe River basin, including both the cascade of impacts in the eco-hydrological system and the socio-economic impacts. There is no standardized framework for considering sectorial interlinkages for determining drought severity (van Loon et al. 2016; Bachmair et al. 2016), and a systematic approach to data collection, the European Drought Impact Report Inventory (EDII, see Stahl et al. 2016), has been orphaned in 2013.

Multisectoral drought impact assessments have shown the value of interlinking natural and socio-economic consequences. Examples include Eklund and Seaquist (2015) on the full impact cascade of drought in Iraqi Kurdistan, Quiroga and Suarez (2016) on income distribution shifts in Spain, Carse (2017) on supply issues at the Panama Canal during the 2015–2016 drought, Vicente-Serrano et al. (2021) on vegetation and agriculture in a Spanish basin and the review of Wlostowski et al. (2022) on nonagricultural socio-economic sectors in the Western US. For Europe, Ionita et al. (2017) assessed the 2015 drought mainly affecting Eastern Central Europe and Southern Germany in detail using meteorological fields, while prominent multisectoral impacts have been addressed by Van Lanen et al. (2016). A recent literature analysis about cascading drought effects included at least the year 2018 of the Central European drought (Niggli et al. 2022).

This study considers both the progression of hydrometeorological impacts in North-Eastern Germany and the socio-economic impacts of the 2018–2019 drought. Another question is how resilient infrastructure and societal conditions will be under future droughts of comparable magnitude, a scenario increasingly likely under climate change—and already looming with 2022 being another drought year in large parts of Europe. Finally, detailed knowledge about all aspects of these extremes is imperative for preparing European societies for future major drought events.

## Materials and methods

### Research domain

**Fig. 1:**
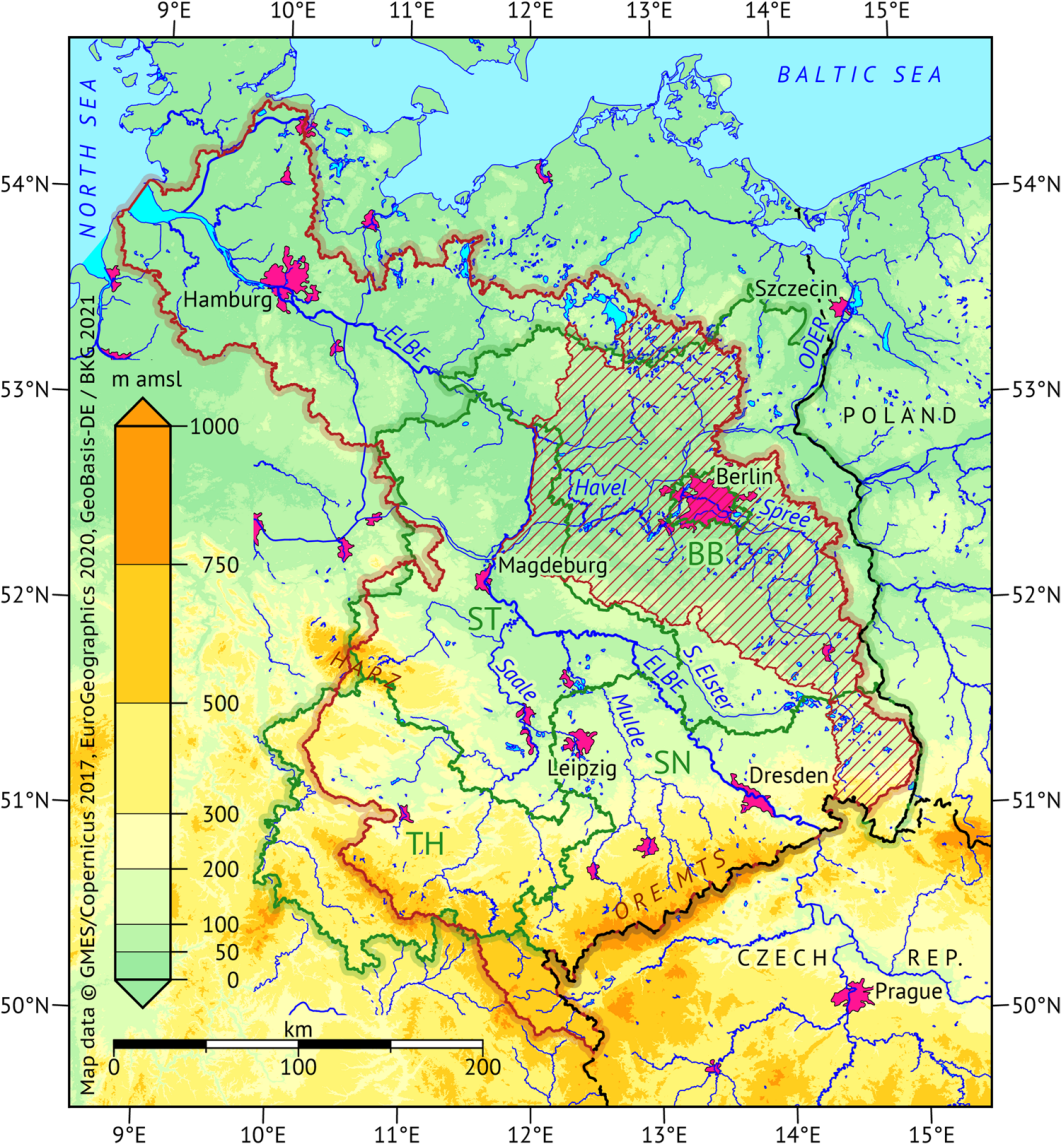
Map of the research domain. Basin boundaries (red brown) with the Havel River subbasin (hatched) extracted from BfG (2020). Data available for administrative units only are regularly analyzed for an aggregate of five federal states (green boundaries): Berlin, Brandenburg (BB), Saxony (SN), Saxony-Anhalt (ST) and Thuringia (TH)

The area of interest is the German part of the Elbe River basin (GEB) covering an area of 97,175 km^2^ (Simon et al. 2005) with a special focus on the Havel River subbasin (Fig. 1). River basins are natural observation units for droughts (and floods) due to the gauged water discharge. Parallel analyses for the Havel River subbasin were motivated by the question of transferability (*pars pro toto* principle) and the fact that drought factors (lower precipitation and average soil water capacity) concentrated here.

As many data are not available for hydrological basins but administrative units, we often combined federal state data from Berlin, Brandenburg, Saxony-Anhalt, Saxony and Thuringia (green boundaries in Fig. 1) as a proxy for the average GEB conditions. A geographical description of the research domain is given in Supplementary section S1.

### Data sources

Basin boundary geodata were obtained from the German Federal Institute of Hydrology (BfG 2020). Other vector geodata, including administrative boundaries, have been provided by Eurogeographics (EGM 2020) and the German Federal Agency for Cartography and Geodesy (BKG 2021). Land surface elevations were taken from EU-DEM v1.1 (Copernicus 2016), and Corine Land Cover (CLC) data for 2018 were applied (Copernicus 2020).

The German Weather Service (Deutscher Wetterdienst, DWD) regularly publishes monthly 1-km gridded meteorological variables over Germany (DWD-CDC 2021a,b,c). The GEB and Havel areas were extracted from these data to serve as the basis for the climate portrait in section S1 and the meteorological drought indices detailed below. Based on a personal request, the DWD also provided daily UV radiation measurements of the station Lindenberg (52°12.5′N, 14°07.1′E).

The agrometeorological model of the DWD, AMBAV (Löpmeier 1993, 1994, n.d.) calculates the potential evapotranspiration according to Penman–Monteith and the actual vertical water fluxes for a sandy loam standard soil. Soil water content is among the modelled variables, and the results can also be readily obtained as monthly gridded data products (DWD-CDC 2021d). Monthly soil moisture index (SMI) maps considering Germany’s diverse soil landscape were obtained from the Helmholtz Centre for Environmental Research in Leipzig (UFZ 2021). Weekly global grids of NASA’s GRACE-Based Shallow Groundwater Drought Indicator in 0.25° resolution were obtained from their dedicated website (NDMC 2022).

Daily river discharge data of the Elbe gauges Dresden and Neu Darchau and the Havel gauge Rathenow UP for the years 1961–2019 had been obtained from the Global Runoff Data Centre (GRDC 2021), Koblenz, Germany. The runoff time series of the Havel River gauge Rathenow UP was extended by the year 2020 on request by the Brandenburg State Agency for the Environment (LfU) on behalf of the Waterways and Shipping Office Spree-Havel (WSA Spree-Havel).

Vegetation status monitoring by the Plant Phenology Index (PPI) was calculated from the Moderate Resolution Imaging Spectroradiometer (MODIS) nadir bi-directional reflectance distribution function (BRDF) adjusted reflectance (NBAR) product (Version 6.0) at a daily time step and 500-m spatial resolution (Schaaf and Wang 2015). Vegetation areas were masked using the CLC 2018 (Copernicus 2020).

For socio-economic data, the electronic data bases of Eurostat (Eurostat 2022) and the German Statistical Offices (DESTATIS 2022a,b; Statistische Ämter 2022b) have been consulted. These were extended by special resources for federal state-level national accounting (Statistische Ämter 2022a) and energy balances (LAK Energiebilanzen 2022).

Environmental data on freshwater use could be obtained via the national environmental accounting (Statistische Ämter 2022c), air pollution data is available from the German Environmental Agency (UBA 2022). Further data points collected from websites or literature are referenced individually.

### Drought severity indicators

Meteorological drought is defined as an extended time period with less than average precipitation. While there are many drought severity indicators, there is no generally accepted rule about the duration or deviation thresholds used to identify drought events (WMO and GWP 2016). Among the most important drought indices are SPI and SPEI:

- SPI: the Standardized Precipitation Index (McKee et al. 1993) was recommended in 2009 by an international WMO workshop as the main meteorological drought index that countries should use to monitor and follow drought conditions (Hayes et al. 2011). The only input is a long time series of precipitation (at least 20 years of observations) from which normalized deviations from period averages are calculated. Typically, index values below − 1 are flagged as drought situations (WMO 2012).
- SPEI: the Standardized Precipitation Evapotranspiration Index (Vicente-Serrano et al. 2010; Beguería et al. 2014) also incorporates, as the name suggests, potential evapotranspiration. This allows consideration of drought intensification through rising temperatures under climate change, a common observation even if there is no long-term trend in precipitation. The calculus and interpretation are similar to SPI. SPEI has been widely recognized and is combined with SPI into the GPCC-DI drought index of the Global Precipitation Climatology Centre (Ziese et al. 2014). The SPI and SPEI time series used for this study have been calculated from area averages of gridded precipitation and temperatures (DWD-CDC 2021a,b) using the SPEI R package (Beguería and Vicente-Serrano 2017).

The original soil moisture index (SMI) of the UFZ drought monitor (UFZ 2021) is a rescaled soil water content modelled daily and nationwide with the mesoscale hydrological model (mHM) for profiles of approximately 1.8-m depth at 4-km spatial resolution (Zink et al. 2016). The rescaled values give the long-term probabilities of lower soil moisture in the respective raster cell and month based on a time series going back to 1951. Hence, the historical SMI time series have a uniform distribution between 0 and 1; values of 0.2 or less indicate a drought condition. To compare regional averages in the Z-score metric of the other drought indices, we transformed the *n* grid cell values of a time step *t*, to standard normal distribution before averaging (Eq. (1)). As the spatial averaging reduced some variance, the time series values were normalized by their respective sample standard deviation (Eq. (2)). This derived index is henceforth named SMI*.

1 $${\text{SMI}}{^{\prime}}_{t}=\frac{{\sum }_{k=1}^{n}{\Phi }^{-1}{\text{SMI}}_{k,t}}{n}$$

2 $${\text{SMI*}}_{t}=\frac{{\text{SMI}}^{\prime}_{t}}{\sqrt{\frac{{\sum }_{t=1}^{T}{\left({\text{SMI}}^{\prime}_{t}-\overline{\text{SMI}^{\prime}}\right)}^{2}}{T-1}}}$$

A GRACE-based groundwater index (GGI) was obtained in the same manner from weekly grids of NASA’s GRACE-based shallow groundwater drought indicator (Houborg et al. 2012; Li et al. 2019). These data were also aggregated to the monthly time scale.

To assess drought severity from the streamflow perspective we calculated the Standardized Streamflow Index (SSI) following the Best Monthly Fit (BMF) approach by Vicente-Serrano et al. (2012) (see section S2.3 for details). For the Havel area, runoff measurements of the Rathenow gauge representing 80.8% of the entire Havel river catchment (including the Czech part) could be used directly for SSI calculation. The GEB discharge contribution had to be approximated from the difference area between the gauged catchments of Dresden and Neu Darchau (78,854 km^2^) which includes the Havel catchment. The average time lag of a flood wave from Dresden to Neu Darchau is 6.5 days, and the distance between the gauges along the river course is 480.84 km which indicates a wave celerity of about 0.9 m/s. Hence, the streamflow contribution of the intermediate area was estimated by subtracting the respectively time-lagged runoff at Dresden from the runoff at Neu Darchau. Negative contribution values around flood peaks were avoided by considering the flood wave dispersion through gamma kernel smoothing of the Dresden time series. The error of this procedure should be less than that of the original gauge measurements. A map with the three gauges and their (difference) catchments is shown in Fig. S9 in the Supplement.

For monitoring drought impacts on vegetation growth, the Plant Phenology Index (PPI) was utilized. While primarily based on daily NBAR reflectance in red and NIR bands using MODIS data similar to other vegetation indices, the PPI calculation (Jin and Eklundh 2014; Karkauskaite et al. 2017) considers canopy geometry and soil reflectance characteristics which makes the index relatively robust to snow and maintains a linear relationship to the leaf area index (LAI). PPI has been used for estimating trends in vegetation phenology (Jin et al. 2019) and is used as a standard productivity and phenology indicator in the recently released European Copernicus high-resolution data sets (Tian et al. 2021). We used monthly PPI aggregates of our focus areas and finally calculated Z-scores (PPI-Z) which can be interpreted as a standardized vegetation health index.

## Results

### Drought severity and progression through natural systems

#### Meteorological drought

**Fig. 2:**
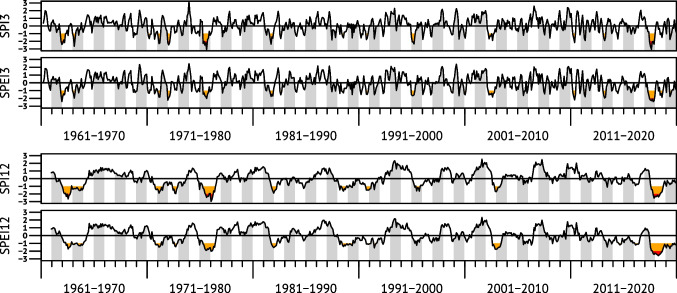
Meteorological drought indices (SPI and SPEI) for the GEB, calculated for 3- and 12-month time scales. The orange and red threshold levels are at −1.0 and −2.0 indicating drought and extreme drought, respectively

Figure 2 shows time series of the meteorological drought indices over six decades for the GEB. A similar image has been obtained for the Havel area (Fig. S6). The upper panel of Fig. 2 shows the results for 3-month time scale; the lower panel was calculated with 12-month time scale.

The top event within the six decades occurred in 2018 and the two following years in which precipitation was still below average (in the GEB 430 mm, 573 mm and 584 mm, respectively, compared to 656 mm a^−1^ in 1991–2020; see also Fig. S2b) and could not compensate the severe loss from soil and groundwater storages. This is correctly captured by the SPEI. Furthermore, SPI shows that the 2018 drought severity was comparable to the two major events in 1963 and 1976, whereas SPEI shows that the 2018 event was the most severe among all events when considering high evaporative demand by elevated temperature.

The 1976 drought was however certainly second in severity within the six decades, and the drought of 2003 ranks third. Notable also are dry starts of two pairs of subsequent years, 1963 and 1964 as well as 1972 and 1973, the dry autumn of 1982, and, especially in the Havel area, extended below-average precipitation in and around 1989.

#### Soil moisture deficit

Like for most areas around the world (Novick et al. 2022), there are no spatially distributed long-term soil moisture measurements for the Elbe basin; respective efforts have been started only recently (Bogena et al. 2022). We therefore resort to modelled data, a customary way to integrate the scattered observation data and obtain a consistent picture over several decades (see Boeing et al. (2022) or Sungmin et al. (2022) for recent developments).

**Fig. 3:**
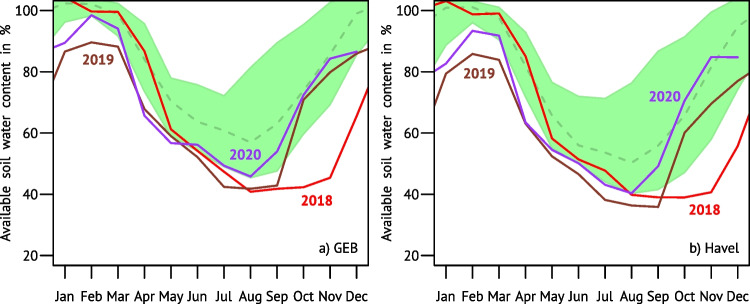
Seasonal cycles of available soil water modelled with AMBAV for a standard soil (sandy loam) of 1 m depth. Regional averages for the GEB (**a**) and the Havel area (**b**). The green areas and dashed lines indicate the 10–90 percentile ranges and median of the years 1991–2020. Data basis: DWD-CDC (2021d)

Soil water contents show time-lagged dynamics, especially towards the deeper layers. For a synthetic 1-m profile of loamy sand under grass, Fig. 3 shows the domain-specific seasonalities for the period 1991–2020 calculated from DWD’s gridded AMBAV product (DWD-CDC 2021d). The percentages in Fig. 3 represent the plant-available water storage relative to the soil-specific maximum (field capacity).

There has been a clear trend towards drier soils in recent years. Especially in May and June the modelled soil became increasingly dry, and the regular autumn recovery was occasionally hampered. After 2018, soil water contents were at extremely low levels as late as November and could not fully recover in the following winter. The lowest February values (brown lines, 89.6% for the GEB and 85.8% for the Havel area) were observed in 2019 as well as the record minimum of 35.9% in the Havel area in September of the same year.

The UFZ Drought Monitor SMI maps showing the end-of-summer situations in 2018 and 2019 are reproduced in Fig. S7. While the northeastern part of the GEB was still not much affected in 2018, 1 year later almost the entire research domain was struck by exceptional soil moisture deficit (observed in less than 2% of the respective months since 1951). The end-of-winter situations (Figure S8) signal that soil moisture did not recover to normal levels in most parts of the GEB even by 2021.

#### Drought propagation and response in vegetation

**Fig. 4:**
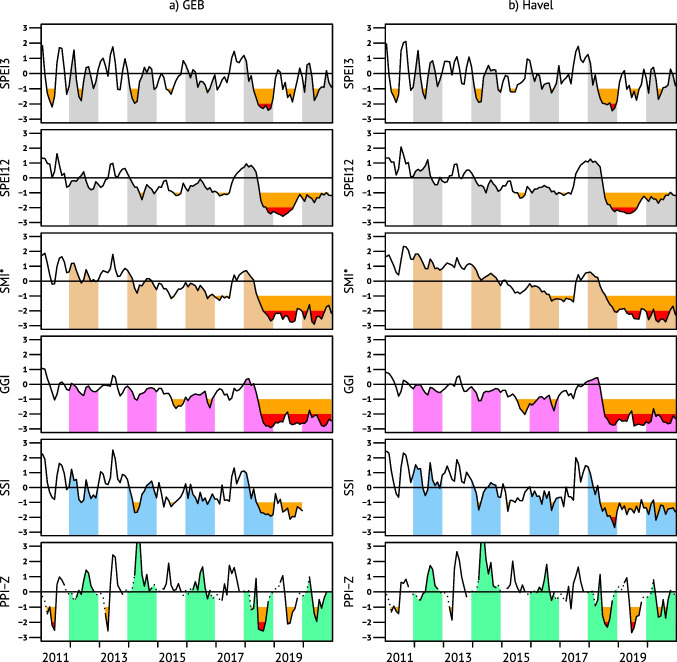
Drought indices for the decade of 2011–2020 for GEB and Havel area. From top to bottom, the 3- and 12-month meteorological SPEI, the Z-score rescaled German Drought Monitor’s Soil Moisture Index (SMI*), the GRACE-based Groundwater Index (GGI), the Standardized Streamflow Index (SSI) and Z-scores of the Plant Phenology Index (PPI-Z; dots denoting winter dormancy period with PPI < 0.05) are depicted. Red and orange colours indicate drought and extreme drought thresholds as in Fig. 2. The 2020 gap in the SSI of the GEB is due to missing runoff data

The drought propagation into the natural systems is shown in Fig. 4 by comparing the area-specific indices for meteorological (SPEI3 and SPEI12), soil (SMI*), groundwater (GGI), streamflow (SSI) and vegetation (PPI-Z) systems over the last decade. Soil water storage became gradually depleted over more or less the entire decade which may explain the extremely low SMI* levels even in 2020. The sustained effect of depleted storages is also visible in the GGI and SSI indices which were almost constantly in (extreme) drought state since the summer of 2018.

Dry conditions in spring during the onset of the vegetation period are a major threat for plants. In 2003, this was the case in many parts of the Elbe River basin in March and April, whereas in 2018 these months were still humid enough, favouring spring vegetation growth that contributed to soil moisture depletion and consequently exacerbated summer drought (Peters et al. 2020). Note however the PPI-Z peak in early 2014 despite the dry spell.

The large evapotranspiration losses in the summer of 2018 prevented usual grassland development even in subsequent years (Reinermann et al. 2019; Kowalski et al. 2022). Consequently, in 2019 and 2020, deeper rooting vegetation was affected as well. Although vegetation response in compound events is generally complex (Bastos et al. 2021), damages were visible in the forests of the Elbe River basin. The stress in canopy development peaked in the summer seasons (see the PPI-Z time series in Fig. 4).

Based on a former European Council regulation (EEC 3528/86) (1986), the federal states publish observed percentages of damaged trees, the most recent of the annual reports considered here are SVUVK (2021), MLUK (2021) SMEKUL (2021b), MULE (2021) and TMIL (2021) (for details of this forest status monitoring (*Waldzustandserhebung*), see Wellbrock et al. (2018)). Time series plots for the five federal states are shown in Fig. S13. In 2019 and 2020, more than 30% of trees in Thuringia and more than 50% in Saxony were significantly damaged. Even in 2021 damage shares remained very high, exemplifying the enormous time lag of drought reaction in trees including compound effects—in this case outbreaks of bark beetles (see below and the following section regarding the socio-economic impacts).

#### Drought effects on wildlife

Regarding drought effects on biodiversity there are only a few sources specifically focusing on the 2018–2019 drought in Eastern Germany. In December 2018 and January 2019, vast amounts of fish died in canal systems near the Elbe estuary. The fishes were dying from aluminium (Al) contamination which damages the gills and inhibits oxygen uptake. Low groundwater levels initiated oxidation processes which led to acidification causing Al dissolution from the clay minerals in the soil. Precipitation events in December 2018 finally transported the contaminated water into the canal system (Möllers n.d.; Völlinger n.d.).

Annual amphibian inventories in the Stechlin-Ruppiner Land Nature Park in Brandenburg showed population breakdowns of about two-thirds for a number of species in 2019 (Naturwacht n.d.). Similar effects have also been reported from Thuringia where small water bodies dried out and spawn clumps of frogs decreased in size. The decline in amphibian populations also affects white storks (*Ciconia ciconia*) which feed on amphibians, especially frogs. In Eastern Germany, the stork population stagnated in recent years while other parts of the country saw certain increases (BAG Weißstorchschutz 2018–2020).

While insect populations are not generally monitored, drought-triggered outbreaks in bark and jewel beetle populations have been reported in conjunction with the severe forest damages caused by the larva living in the bark of trees (SMEKUL 2021a,b; TMIL 2021). The drought also promoted other insects, e.g. the dragonfly species *Crocothemis erythraea* and the rose beetle *Oxythyrea funesta.*

Finally, the population of wolves (*Canis lupus*) is closely monitored since their return to Germany in the year 2000. Their main distribution is inside the GEB, and their numbers increased almost linearly from 77 packs in the observation year 2017/2018 to 157 packs in 2020/2021 (DBBW 2021). While the speed of their actual spread does not seem to be drought-affected at all, their specific sensitivity may become apparent in future years after the population will have stabilized on a higher level.

### Socio-economic sector-specific drought impacts

We assessed the socio-economic impacts by aggregates of the statistical classification of economic activities in the European Community (NACE) Rev. 2 (Eurostat 2008), indicated by capital letters. The socio-economic impact assessment is mainly based on official data from the Statistical Offices of Germany, detailed references are given with the individual results; the same holds for further literature sources.

#### Agriculture, forestry and fishing (NACE section A)

**Fig. 5:**
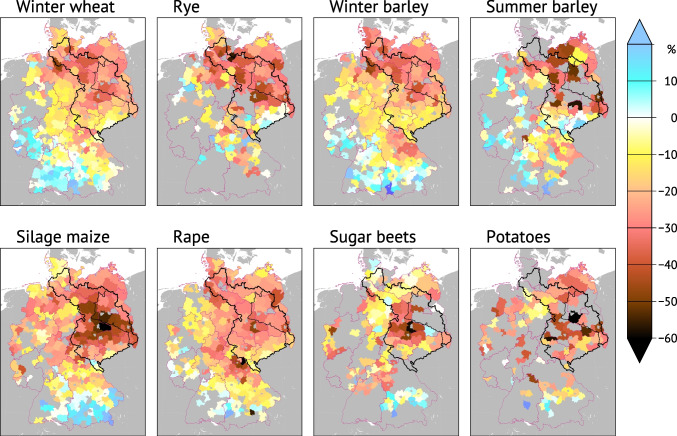
Crop yields in 2018 relative to the 2012–2017 averages in German district-level administrative units; grey areas indicate missing data. GEB and Havel area are outlined in black. Data source: Statistische Ämter (2022b). Map geometries © GeoBasisDE/BKG

In 2018, GEB crop yield averages dropped by 20–40% compared to their pre-drought levels (cf. Figs S10 and S11). The maps in Fig. 5 show that the GEB was indeed the regional centre of yield losses in 2018. Some farmers experienced complete failure of their crops and consecutive personal hardships which remain unaccounted by the integrated economic impact analysis. Still moderate losses were reported for wheat and barley (because they are preferentially planted on richer soils with higher available water capacity), while maize (corn and silage) experienced the most extreme losses.

Although many meat and dairy farmers had to buy expensive extra fodder from abroad, the drought is not visible in livestock numbers or slaughtering rates. To quantify the economic impact on the agricultural sector as a whole, we inspected the developments of gross value added (GVA) and workforce in the agricultural sector (Fig. S12). Over the last decade, both variables have been in continuous decline, not only in terms of relative importance but also their absolute economic contribution. In terms of inflation-adjusted 2015 euros, the sectorial GVA peaked at 4.267 billion in 2014; hence, the 2017–2018 drop from 3.515 to 2.746 billion looks more like a random fluctuation, a loss of merely 0.2% for the entire regional economy with its 2018 GVA of 403.9 billion euros (Statistische Ämter 2022a). The monotonous decline in agricultural workforce, − 14% between 2011 and 2020, was however accelerated by the drought. In 2020, the COVID-19 travel restrictions prohibited many seasonal workers from taking up their jobs aggravating the downtrend. This may explain why the GVA remained very low in 2020 despite a partial recovery of yields.

**Fig. 6:**
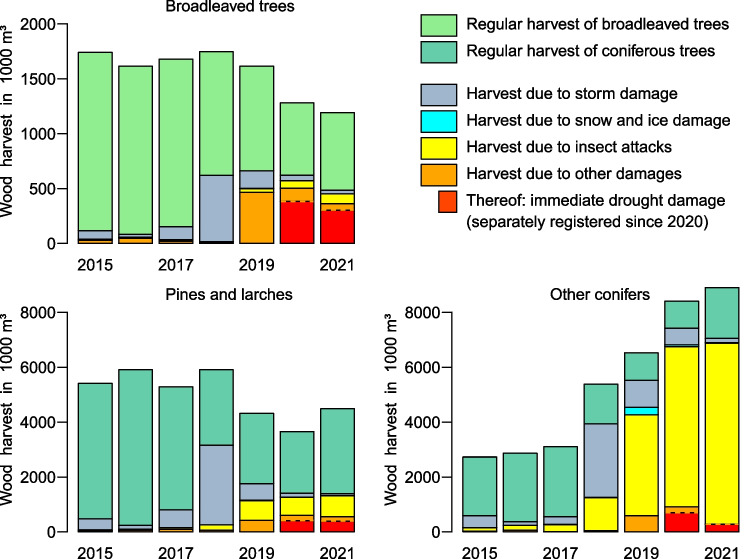
Recent annual wood harvests in the five-state aggregate separated by species groups. Indicated are also cutting volumes caused by actual damages. Data source: DESTATIS (2022a)

According to CLC 2018 (Copernicus 2020), 28.3% of the GEB surface is forested, and a forest share of 29.7% was officially determined for the area of the five federal states Berlin, Brandenburg, Saxony, Saxony-Anhalt and Thuringia (DESTATIS 2021). The drought shocks in forestry can be accessed through the official harvest assessments (DESTATIS 2022a) which are aggregated and visualized for the five states in Fig. 6.

Before 2018, approximate annual harvest rates amounted to 1.7 million m^3^ of wood from broadleaved and 8.5 million m^3^ from coniferous trees, the latter dominating in Eastern Germany. Until 2018, damaged wood accounted for minor shares in the harvest statistics. The situation changed when a series of gales struck in October 2017 (Haeseler 2017; Haeseler and Lefebvre 2017) and January 2018 (Haeseler et al. 2018). In 2018, broadleaved trees were affected most because their leaves are prone to high transpiration losses, and they are more frequent in the comparatively dry lowlands.

An indirect drought impact is the growth of damages caused by insects, especially in “other conifers” in Fig. 6. These are typically spruce trees in mountainous areas attacked by bark beetles. The current bark beetle infestations are the most severe since those of the 1980s when many high-altitude areas in the Ore Mountains had been cleared. Especially for spruce forests, the general damage situation has never been worse (BMEL 2021), and complete spruce diebacks occurred in some locations; the situation slightly alleviated in 2021 (SMEKUL 2021b).

There were probably positive short-term economic impacts in the forestry sector: more wood was cut down and sold at relatively stable price levels. However, reforestation and substantial losses in wood capital will most probably outweigh those surpluses in the long run (Seintsch et al. 2020). Möhring et al. (2021) suggest losses of 12.750 billion euros from the 2018–2020 damages for all of Germany; neglecting regional differences in hectare values and drought severity this would mean roughly 3 billion euros within the GEB.

Forest fires affected more than 21 km^2^ in 2018 and 15 km^2^ in 2019 in the five federal states (Table S4). The situation eased in 2020 with only about 1.7 km^2^ which is below the average of the non-drought years 2015–2017 (BLE 2016–2021). For 2018 economic losses in Saxony, Saxony-Anhalt and Thuringia were estimated at 1.65 million euros, more than tenfold the non-drought year average.

Inland fisheries are a minor economic activity; the last official survey (*Binnenfischereierhebung*) dates back to 2004. Based on figures collected by Brämick (n.d.), we estimate a gross revenue estimate of about 50 million euros per year. Fish production was also reduced, numbers reported for single species and federal states suggest general losses of 20–30% in quantity (for more details, see section S3.3).

#### Mining and quarrying, manufacturing and utilities (NACE sections B–E)

No news reports about reduced industry production due to water shortages could be found for the Elbe region. The big industrial water demand—954 million m^3^ for mining and manufacturing and 492 million m^3^ for private households in the five state aggregate in 2016 (Statistische Ämter 2022c)—could be constantly met, at least where no cooling was involved. Permittances for the discharge of warm water were reduced in 2018 (as in the hot summers of 2003 and 2006; IMAA 2019), but in the GEB this did not affect major thermal power plants.

Between 2015 and 2019, the annual gross electricity production in the five-state aggregate ranged between 136.0 and 145.9 TWh year^−1^, the upper boundary reached in 2018, the first drought year (LAK Energiebilanzen 2022; data for 2020 not yet available). As there are no active nuclear power plants in the GEB, the lignite-fuelled plants in Lusatia (Brandenburg and Saxony) alone deliver nearly half of this electricity, approximately 65 TWh year^−1^ (Fig. S14). Koch et al. (2014) analyzed the problems for electricity production in the Elbe basin under a dry climate scenario, and the reality of the recent drought seems to follow the projections. A cooling water shortage problem was however limited to small stations in the city of Berlin; the big lignite-fuelled power plants near the open-cast mining areas were not affected at all because the groundwater uptake from the mining areas delivered enough water with suitable temperatures (approx. 11°C). Furthermore, these power plants, namely Boxberg, Jänschwalde and Schwarze Pumpe, use water-saving closed-loop systems with cooling towers as heat exchangers which makes them practically fully resistant against heat and drought.

While in many GEB districts unauthorized water withdrawals from rivers and lakes are regularly forbidden in each summer since 2019, actual tap water supply problems affected only very few municipalities. The village of Harsefeld, approximately 25 km west of Hamburg, was confronted with pipe pressure breakdowns in the summers of 2018–2020. Rickert et al. (2022) searched German press reports about private wells running dry in the years 2018–2020: 115 respective news items were found of which 44 referred to locations in Saxony.

#### Transportation (NACE section H)

The hauling capacity of the German transport sectors kept a relatively stable level between 2016 and 2020 with annual values ranging between 671.8 (2020) and 697.9 (2019) billion ton-kilometres. Road and rail had quite stable transport shares of about 71% and 18.5%, respectively (DESTATIS 2022b).

Marginally affected was the hauling capacity on German waterways, dropping from 55.5 billion ton-kilometres in 2017 to 46.9 billion ton-kilometres in the first drought year (DESTATIS 2022b). Considering the general downtrend in inland navigation, the 2018 figure fell short of the general trend by 6.9 Gt km (− 13%), and in 2019 by 2.0 Gt km (− 4%) (see Fig. S15). Regional figures for the GEB are hard to obtain (see section S3.8 for details), but the Elbe River upstream from the big international port of Hamburg is rather unimportant for hauling, not only compared to Hamburg’s offshore relations but also to other inland waterways such as the Rhine River (Table S6).

#### The service sector (NACE sections J–Q)

This section refers mainly to office work or indoor activities: (J) information and communication; (K) financial and insurance activities; (L) real estate activities; (M) professional, scientific and technical activities; (N) administrative and support service activities; (O) public administration and defence, compulsory social security; (P) education and (Q) human health and social work activities.

No outages in electricity and tap water supply are a good precondition for indoor work, only air conditioning is still more of an exception than the rule in the GEB, and many indoor work places became uncomfortably hot in the summers of 2003, 2018 and 2019. This problem is reflected in recent demand peaks for air conditioning sets as seen in the imports and exports visualized in Fig. S17. Unfortunately, no reliable estimation of a temperature–productivity relationship is possible yet; a brief review of the research gap is given in section S3.10.

**Fig. 7:**
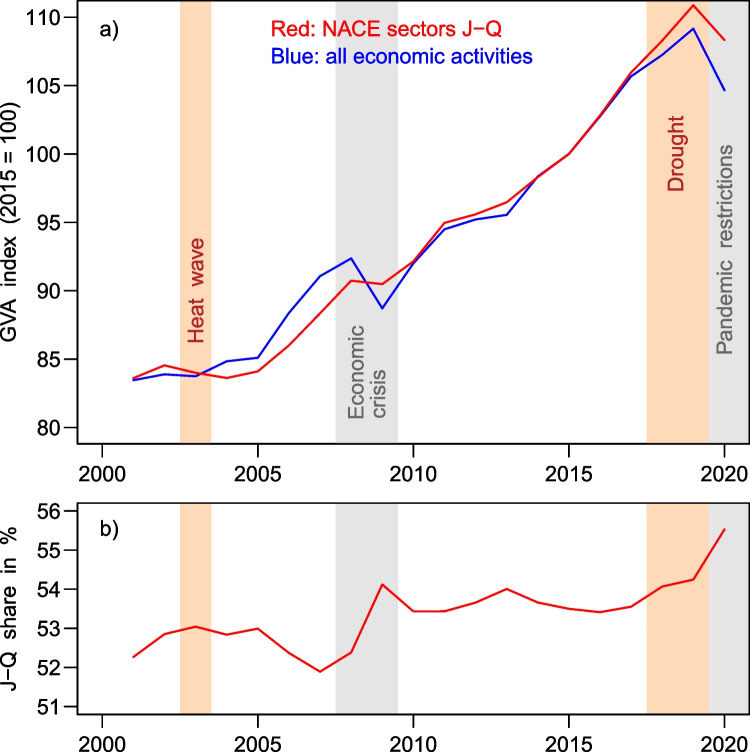
**a**) Relative developments of the gross value added for the narrower service sector and the entire economy in the five-state aggregate. Index values, chained and corrected for inflation. **b**) Share of the narrower service sector in the total GVA of the five-state aggregate, calculated from absolute GVA figures in actual prices. Data source: Statistische Ämter (2022a)

Econometric time series on the service sector’s productivity in the five-state aggregate are shown in Fig. 7: comparing the sector specific to the total GVA in Fig. 7a shows primarily a parallel growth of services with the entire economy. A strong resilience against the financial crisis of 2008/2009 is also visible. Furthermore, neither any influence of the 2003 heat wave nor of the 2018–2019 drought on the service sector can be identified. A similar picture is conveyed from the percentages of this sector in the total economy shown in Fig. 7b. The services share even reached a new record during the recent drought, and the long-term trend of slowly deteriorating importance of the primary and secondary sectors seems to persist.

### Drought impacts on human health

The region-specific drought-related health risks were heat, ultra-violet (UV) radiation and air pollution. The German Weather Service (DWD) issues daily heat alerts based on the concept of perceived temperature (Staiger et al. 2012). In 2018 and 2019 there were 1326 and 978 official warnings, respectively, for strong or extreme heat stress for districts in the five-state aggregate. Considering the annual warnings in the years 2011–2021 whose median is at 687, these numbers ranked first and third. Only in 2015 was a similar level reached with 1153 warnings, but as the warning thresholds had been altered over time comparability may be limited. The heat-related death toll in Berlin and Brandenburg was remarkably high in 2018 with about 700 fatalities, two-thirds of them aged 80 and above (Axnick 2021). This analysis also showed that not only extreme temperatures but also the duration of heat waves is decisive for mortality. In that respect, 2019 was a less severe year despite the hottest summer since 1985 (Axnick 2021).

There are only a very limited number of stations observing UV radiation. We could obtain daily measurements of UV indices (a dimensionless measure of the energy flux in the sunburning action spectrum on the ground around noon, WHO 2002) and sunshine duration from the DWD station Lindenberg, approximately 60 km southeast of the city centre of Berlin, for the years 2015–2021. We restricted our analysis, visualized in Fig. S18, to the summer months June–August as they have the highest radiation intensities and are most critical due to open-air activities and skin-exposing summer clothing.

There is no empirical correlation between UV stress and sunshine duration per month due to the variability of the stratospheric ozone layer and high intraday variation of the solar zenith angle. Consequently, the above-average sunshine of the drought years 2018 and 2019 is generally not mirrored in the UV indices whose monthly averages fall into the usual ranges between 6 and 7 for June and July, and between 5 and 6 for August. Only June 2019 was a positive outlier in both series with a UV index of 7.46 and 369.9 sunshine hours (Fig. S18). Given the linear scale of the index, the sunburn hazard in the drought years was only marginally elevated compared to other years.

Higher UV radiation is however also harmful in combination with nitrogen oxides in the air: this combination triggers chemical reactions producing ozone near the surface. Ozone stresses the human respiration system which leads to symptoms like coughing, headache and restricted pulmonary function (UBA 2020). Ozone levels did peak during the drought years 2018 and 2019. Measurement stations in the five federal states counted on average 37 and 24 days exceeding the critical 8-hour level of 120 μg/m^3^ O_3_ in 2018 and 2019, respectively, compared to an average of 17 days per year in 2010–2017 (UBA 2022).

## Discussion

Our analysis of the 2018–2019 drought in the German part of the Elbe River basin showed strong impacts on regional hydrology and vegetation, especially crop production and forestry, but few consequences for other sectors of the economy. The drought perturbed the system, but not beyond its current stability domain; this reminds of a global ecosystem analysis by Zhang et al. (2019) who found plant ecosystems surprisingly insensitive against droughts. Zhang et al. however only focused on gross primary productivity or above-ground net primary productivity in single drought years; other effects like potential species recompositions or consequences of extreme droughts were left to further research.

We observed a typical pattern of drought with cascading eco-hydrological consequences. This so-called drought propagation has gained scientific interest in recent years, but studies usually stop at hydrological drought (Wang et al. 2016; Peña-Gallardo et al. 2019; Ma et al. 2021; Ho et al. 2021) or at agricultural drought (Zhu et al. 2021). A closer look at Fig. 4 reveals not only temporal lags but also nonlinearities in the ecohydrological system which call for closer investigation, especially PPI-Z peaking in 2014—probably caused by a combination of above-average radiation with soil moisture and shallow groundwater still on relatively high levels—or exposing distinct summer troughs in 2018–2020.

The extremely negative GGI values in Fig. 4 are confirmed by historically low groundwater levels observed in the years 2019–2021 at hundreds of sites in Brandenburg (LfU 2022). This marks the preliminary end point of negative trends observed since the 1980s. The long-term groundwater losses are primarily caused by climate change; in most places groundwater extractions for tap water and industrial needs are still way below the natural recharges (LfU 2022). It may be noted here that the concentrated water demand of the city of Berlin is mainly fulfilled by river bank filtrate and is fully returned into the Spree and Havel rivers through wastewater treatment plants. Even in the summer of 2019, the inflows to Berlin practically equalled the outflows from the city (SUVK 2021).

A reason why interlinkages between natural and socio-economic drought consequences are rarely studied might be that, especially in developed countries, industrial production and services seem quite insensitive to drought (Islam and Hyland 2019; Damania et al. 2020)—just as observed in the GEB. Climate risk awareness before 2018 was only well established in the forest sector where long-term programmes for shifting the tree species composition from coniferous monocultures to resilient mixtures with high broadleaf shares had already been initiated about the year 2000 (e.g. MLUK 2004). The positive effect of deciduous trees on groundwater recharge has come into focus only recently, though.

In other sectors, climate risk awareness led a niche existence in all parts of Germany. For a study about climate adaptation in enterprises and municipalities (Mahammadzadeh et al. 2013), industry representatives were asked for their risk perception. Heat was an issue of concern for only 23%, and drought for only 13% of those enterprises which generally cared about climate change. Frost, storms and floods were more frequently named. Higher climate risks were anticipated for the future (2030) with heat being the highest concern (46%). Meanwhile, these concerns can be rated realistic given the peaks in air conditioning demand and heat mortality.

Lühr et al. (2014) focused on possible supply chain disruptions. In procurement, extreme heat can cause traffic disruptions (damaged traffic lanes, higher probability of accidents), and drought often limits navigation along rivers—the latter also realized in the current drought. Process risks include reduced employee productivity, problems in cooling of IT components and workplaces, and heat damage to stocked goods. Due to the international interlinkage of supply chains, the risks are not confined to the drought region. Lühr et al. (2014) see the metal industry as especially drought sensitive because of its high water and energy demand—this did not however become apparent in the 2018–2019 drought.

Virtually, the same points of concern regarding drought and manufacturing were highlighted by Buth et al. (2015), another national study. The authors also made some efforts to differentiate the risks spatially albeit without giving a clear picture for the Elbe region. For the Dresden area Auerswald and Vogt (2010) identified water or energy-intensive sectors of manufacturing. The result, reproduced in Table S5, should be generally transferable to the whole GEB. Interestingly, paper making or printing was not classified as energy sensitive, albeit paper mills and rotation presses would immediately stop without electricity. The same holds for the tools used in manufacture of machinery equipment, a sector named economically important for the Dresden region but neither classified as energy nor water sensitive.

Certain enterprises will likely be confronted with water shortages in the coming decades. The recently opened Tesla factory at Grünheide near Berlin has been criticized for its planned water demand of 3.6 million m^3^ per year. This is just a small fraction of the entire manufacturing sector demand across the region, but the problem is that the demand needs to be fulfilled onsite.

It is striking that in contrast to the uncomfortable scenarios of Mahammadzadeh et al. (2013), Lühr et al. (2014) and Buth et al. (2015), the most intensive regional drought event in centuries did not really hamper the secondary and tertiary sectors of the economy. The service sector in particular seemed to be resilient to external shocks including economic crises, probably because many service jobs are permanent positions with fixed standard wages (especially in the public administration), and their GDP relevance is therefore more or less independent from their actual productivity.

Meanwhile, at the time of writing (end of 2022), another European drought year has materialized, and stability boundaries are tested anew, probably with increased vulnerabilities (Hughes et al. 2019). In our case study region, ecosystem degradation is currently being observed and some local ecosystems destroyed with impacts that may last for years. This includes burnt forest areas in Saxony and an exhaustive fish kill in the Oder River, the neighbouring stream along the Polish border. While both cases are not directly caused by the drought they have at least been enabled by the extreme meteorological conditions. This is consistent with our findings from 2018 and 2019 where there were significant forest fires and fish kills, and considerable forest diebacks since.

## Conclusion and outlook

The 2018–2019 extreme drought in the German part of the Elbe basin exemplified the cascading effect of drought propagation in natural systems (with lagged but locally fatal consequences for vegetation and wildlife), caused significant economic damages in agriculture and forestry but only modestly affected the secondary and tertiary economic sectors. Tap water supply was generally continuously maintained; however, the associated summer heat caused health issues and excess mortality.

This cross-sectoral analysis demonstrates drought-related interdependencies between the natural and socio-economic systems which are understudied but will become more significant with increased frequency and duration of droughts. Emerging evidence on the link between anthropogenic climate forcing and drought (e.g. Chiang et al. 2021) highlights the necessity to increase preparedness for future climate and drought situations across Europe.

With future intensification of droughts under climate change (Ionita et al. 2022), the danger of irreversible drought damages to ecosystems (tipping into new stability domains; e.g. with altered, less diverse species compositions and permanently reduced net primary production) will grow. Driven by the positive temperature trend, evapotranspiration is bound to increase over the next decades. Respective scenarios from 10 years ago (Conradt et al. 2012) are still valid regarding trend directions. This is confirmed by other studies (Horton et al. 2016) as well as by recent climate scenario assessments (Spinoni et al. 2018; Hari et al. 2020; Zhao and Dai 2022) which might still underestimate already observed drought trends (Piniewski et al. 2022). The ecosystems of the Elbe basin should be closely monitored for indications of catastrophic developments, for instance by a spatial extension of the TERENO field observatory in North-Eastern Germany (Heinrich et al. 2018).

Monitoring is also key in the socio-economic system. Blauhut et al. (2022) asked stakeholders across Europe about their perception of the 2018–2019 drought and the drought risk management in their countries. Germany was among the drought-aware but least prepared countries with no management plan in place. While most stakeholders supported the idea of a EU drought directive, the question remains how the complexity of drought effects and other tensions in the regional socio-economic fabric can be addressed.

For instance, given the current (end of 2022) multifaceted challenges in Central Europe, such as price inflation in the Eurozone or insecure food and energy supplies, any socio-economic crisis cannot not easily be attributed to drought alone, save socio-economic disruptions are likely to become more frequent and more damaging under future droughts. To avoid worst-case scenarios like a blackout (Petermann et al. 2011), real-time monitoring and early warning systems for drought and other stress factors in both natural and societal systems should be further developed together with appropriate action plans.

## Supplementary Information

Below is the link to the electronic supplementary material.

Supplementary file1 (PDF 3174 KB)
