# Supplementary material for: Cross-sectoral impacts of the 2018–2019 Central European drought and climate resilience in the German part of the Elbe River basin
Source: Reg Environ Change. 2023 Feb 1;23(1):32. doi: 10.1007/s10113-023-02032-3 (PMC9890441; doi:10.1007/s10113-023-02032-3)
Supplement: Supplementary file 1 — Supplementary file1 (PDF 3174 KB) [file 10113_2023_2032_MOESM1_ESM.pdf]

Supplement to
"Cross-sectoral impacts of the 2018–2019 Central European drought in the German part of the Elbe River basin"

Regional Environmental Change

Conradt T (Potsdam Institute for Climate Impact Research, conradt@pik-potsdam.de),
Engelhardt H, Menz C, Vicente-Serrano SM, Alvarez Farizo B, Peña-Angulo D, Domínguez-Castro F, Eklundh L, Jin H, Boincean B, Murphy C, López-Moreno JI

Contents

S1 Geographical description of the German Part of the Elbe River basin – S1
S2 Drought indices – S9
S3 Aspects of the socio-economic drought impacts – S13

S1 Geographical description of the German Part of the Elbe River basin

S1.1 Extent and physical features

The German part of the Elbe River basin (GEB) covers an area of 97,175 km², 65.5 % of the entire basin (Simon et al. 2005). The Elbe River originates from the Czech Republic where practically the complete upstream third of its basin is located, very small parts of the basin belong to Poland and Austria. Southern German splinter areas of the upstream part are excluded for this assessment, leaving a contiguous area of 96,864 km² (own GIS analysis) loosely enclosed by the geographical coordinates 8°30′E–15°05′E and 49°45′N–54°30′N, see Fig. 1 in the main document.

Some analyses are confined to the Havel subbasin: 23,790 km² of its total area of 23,860 km² are located in Germany (FGG Elbe 2021); to ease readability, "Havel area" henceforth only refers to the German part unless stated otherwise. The mean runoff of the Elbe River at the Czech–German boundary observed during the 20th century was 311 m³/s, and the discharge into the sea was estimated at 861 m³/s (Simon et al. 2005).

With the exception of the the Ore Mountains of late Paleozoic origin surface geology of the GEB domain is characterized by deep sediments ordered by a sequence of glacial series. In the southern and western parts these sediments are topped by loam and loess layers while the central part including most of the Havel area is largely characterized by poor, sand dominated soils. Soils with higher water storage capacity can be found again in the loamy moraine regions of the most recent (Weichsel) glaciation at the north-eastern edge of the GEB or in the silty marshlands around the Elbe estuary.

Land use is dominated by non-irrigated agriculture (43 %), pastures (15 %) and coniferous forests (21 %). Such managed ecosystems – which are, especially on the sand-dominated soils, strongly affected by drought events – cover more than 85 % of the region (Copernicus 2020; see Fig. S1 and Table S1 for details). Built-up areas account for about 8 % of the land demand, and their share rises slowly but continuosly at the expense of agricultural areas, a trend expected to sustain at least in the vicinity of the big cities (Hoymann et al. 2016; Maretzke et al. 2021).

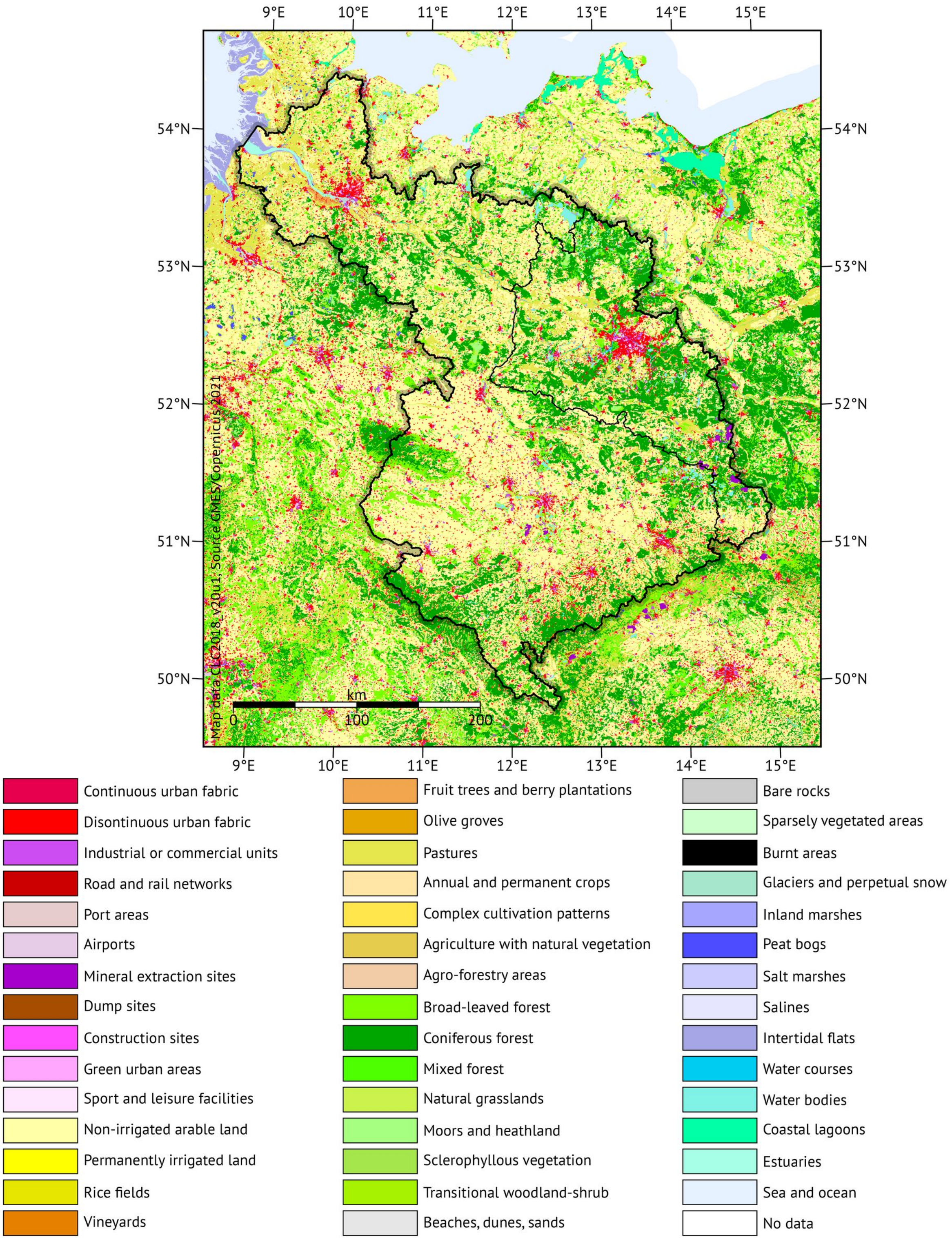

Figure S1: CLC 2018 version v.2020_20u1, downloaded in March 2021. Source: Copernicus Land Monitoring Service, part of the European Earth monitoring programme (GMES).

Table S1: Distributions of land use classes according to CLC 2018 (Copernicus 2020). Included are categories whose coverage exceeds 0.1 percent of the respective basin area. Dominating categories are coloured.

CLC category		GEB		Havel area	
Code	Land use	km²	%	km²	%
111	Continuous urban fabric	155.80	0.16	48.34	0.20
112	Discontinuous urban fabric	6,001.18	6.20	1,535.18	6.46
121	Industrial or commercial units	1,286.06	1.33	345.39	1.45
124	Airports	109.32	0.11	38.85	0.16
131	Mineral extraction sites	379.04	0.39	175.93	0.74
141	Green urban areas	139.00	0.14	53.69	0.23
142	Sport and leisure facilities	547.51	0.57	169.72	0.71
211	Non-irrigated arable land	41,400.68	42.74	7,223.88	30.39
222	Fruit trees and berry plantations	290.18	0.30	26.92	0.11
231	Pastures	14,933.45	15.42	3,526.90	14.83
243	Agriculture with natural vegetation	253.35	0.26	36.41	0.15
311	Broad-leaved forest	5,078.86	5.24	1,013.53	4.26
312	Coniferous forest	20,740.28	21.41	7,829.18	32.93
313	Mixed forest	1,572.77	1.62	480.68	2.02
321	Natural grasslands	290.65	0.30	71.48	0.30
322	Moors and heathland	425.85	0.44	214.59	0.90
324	Transitional woodland-shrub	665.75	0.69	218.19	0.92
411	Inland marshes	114.60	0.12	65.43	0.28
423	Intertidal flats	150.18	0.16	–	–
511	Water courses	213.63	0.22	50.58	0.21
512	Water bodies	1,434.70	1.48	606.02	2.55
522	Estuaries	305.50	0.32	–	–
XXX	Other	375.93	0.39	43.86	0.18
	TOTAL	96,864.27	100.0	23,774.75	100.0

S1.2 Climatology

According to the German Weather Service (DWD-CDC, 2022a,b,c) the 1991–2020 averages of the air temperature at 2 m above ground were 9.4°C in the GEB and 9.7°C in the Havel area. There were upward trends over the last decades, a linear approximation since 1961 yields a regional warming rate of 0.35 K per decade (Fig. 2a). Precipitation varied about 656 mm per year (586 mm/a in the Havel area) and did not expose a significant trend, but the drought years 1976, 2003 and 2018 are clearly visible in Fig. 2b. Sunshine durations however increased with the temperatures (Fig. 2c): The trend lines indicate a surplus of 39.3 hours per decade. The 1991–2020 averages amounted to 1682 sunshine hours per year in the GEB and 1738 h/a in the Havel area.

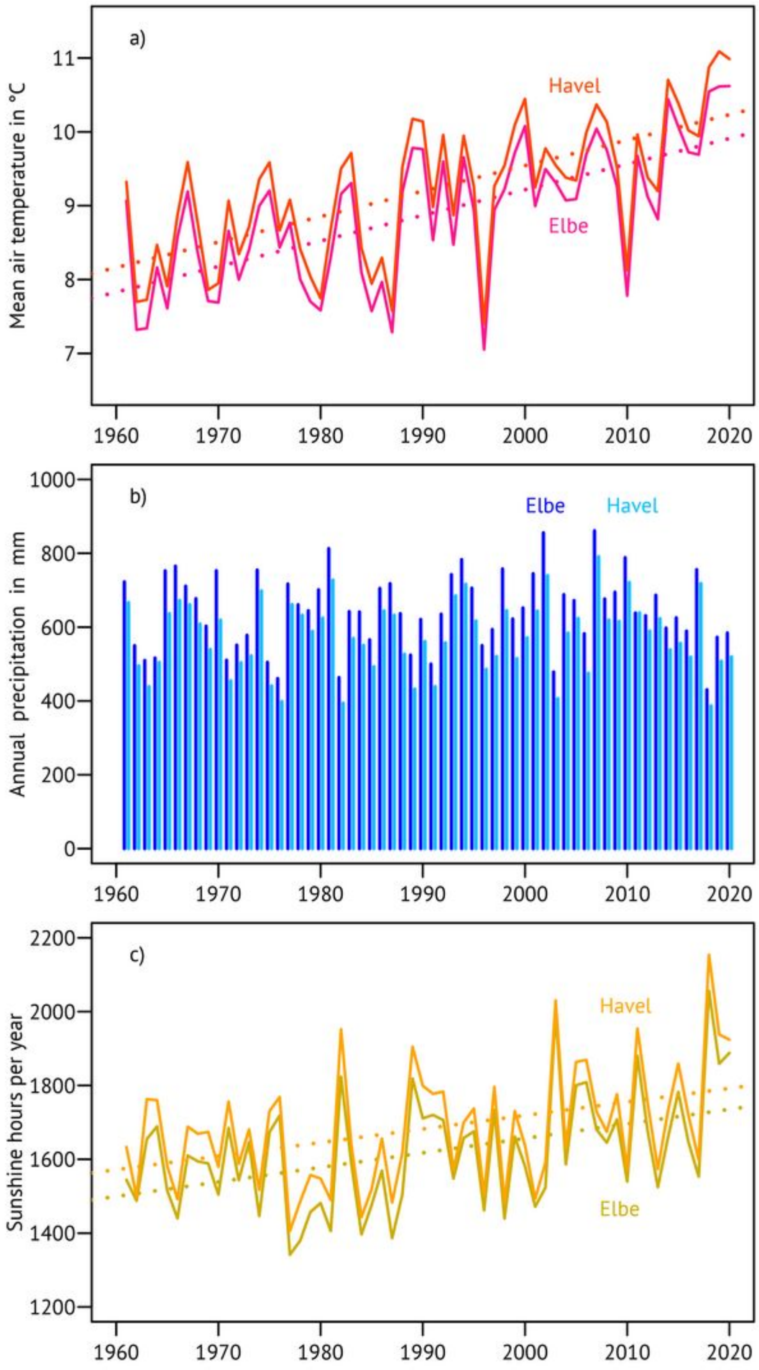

Figure S2: Annual domain averages of air temperature, precipitation, and sunshine hours. Extracted from monthly grid data (DWD-CDC 2022a,b,c)

Since the 1970s the average air temperatures increased at about the double rate compared to the global average (Gulev et al. 2021). The notable increase in sunshine can at least partly be attributed to anthropogenic brightening (Wild 2014; Wild et al. 2021). The Havel subdomain is a little warmer, drier, and sunnier than the GEB as a whole owing to the lower average elevation (82 m less) and a more continentally located centre of gravity. This effect can also be seen in the isohyet map in Fig. S3: Besides the mountains, the coastal region near the Elbe estuary receives above-average precipitation. The map also shows the "rain shadow" of the Harz mountains, this area with spots receiving less than 500 mm in an average year is also the driest region of Germany.

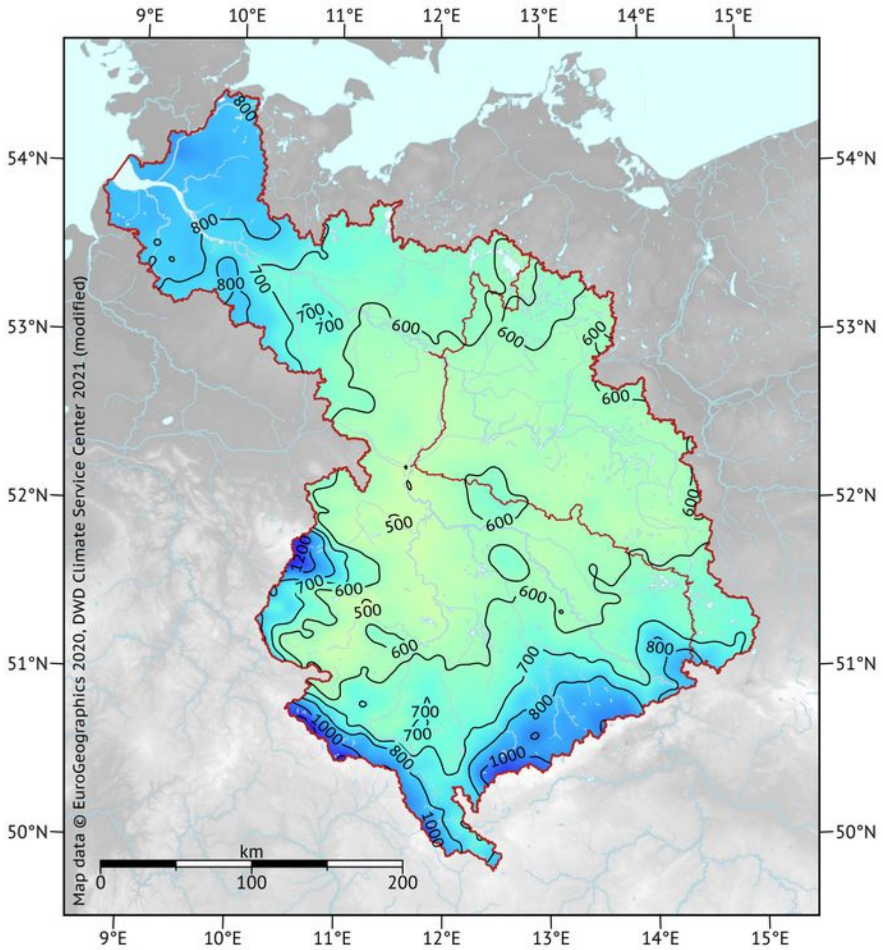

Figure S3: Isohyet map of the German Elbe basin for the climate normal period 1991–2020. Data source: DWD-CDC (2022b).

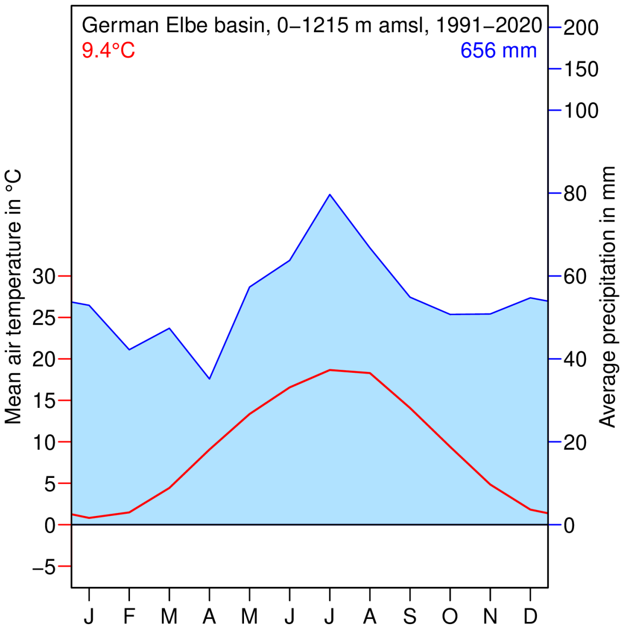

Figure S4: Walter–Lieth climogram for the German Elbe basin, climate normal period 1991–2020. Data source: DWD-CDC 2022a,b.

Regarding the seasonality in the 1991–2020 period (Walter–Lieth climogram in Fig. S4), the coldest month was January with 0.8°C, the warmest July with 18.7°C, which means an average annual amplitude of 17.9 K. The driest month was April with 35.2 mm of precipitation, and the wettest July with 79.7 mm. A substantial share of the summer precipitation is generated in convective clouds; thunderstorms, hail, and heavy rain events occur most frequently during the hot season.

According to the Köppen–Geiger climate classification (as used by Kottek et al. 2006) the GEB largely falls into the Cfb zone: warm temperate, fully humid, with warm summers (warmest month between 10°C and 22°C); only the highest elevations of Harz and Ore Mountains have average January temperatures below −3°C and therefore belong to the respective snow climate Dfb. The Köppen–Geiger classification as "fully humid" is rather simple: The driest summer month is required to have more than 40 mm precipitation on average, and there must not be a strong imbalance of summer exceeding winter precipitation.

A more sophisticated aridity index (AI) was presented with the UNEP World Atlas of Desertification in 1992, now available in its third edition (Cherlet et al. 2018): $AI = (\Sigma_{30} (P_i / ETp_i)) / 30$. In plain text, the index is the average of the 30 annual fractions of precipitation divided by potential evaporation within a climate normal period. Using gridded AMBAV (Penman–Monteith) calculations of potential grassland evapotranspiration (DWD-CDC 2022d), the Elbe region had an AI of 1.06 in the period 1991–2020, and the Havel area AI reached 0.90; both values are considered humid ($0.65 < AI < 0.5$ is considered subhumid, semi-arid is defined by $0.2 \leq AI < 0.5$, and AI values below 0.2 are arid with $AI < 0.05$ classified as hyper-arid).

S1.3 Population

Assuming homogeneous population densities within each of the 2529 municipalities overlapping the GEB, there were 18.143 million residents in the domain on 31 December 2020 (5.842 M in the Havel area). Especially Berlin attracted many people during the 2010s from in- and outside Germany: Between the 2011 census and the end of 2020 the German capital grew from 3.292 to 3.664 million inhabitants, thus contributing 71 % to the population growth in the GEB in this decade (Statistische Ämter 2014; DESTATIS 2021; BKG 2021).

Looking back into the 1990s and the first decade of the 21st century there was an exodus of younger people from the more rural regions of Eastern Germany who saw their employment perspectives deteriorate after the old industries of the former socialist economy had been closed down. Between 1994 and 2017, most districts in Eastern Germany lost 10–25% of their population while similar relative increases concentrated around the big cities of Hamburg and Berlin. Albeit the net out migration has meanwhile come to a halt, further population losses from the rural areas are expected until 2040 (Maretzke et al. 2021).

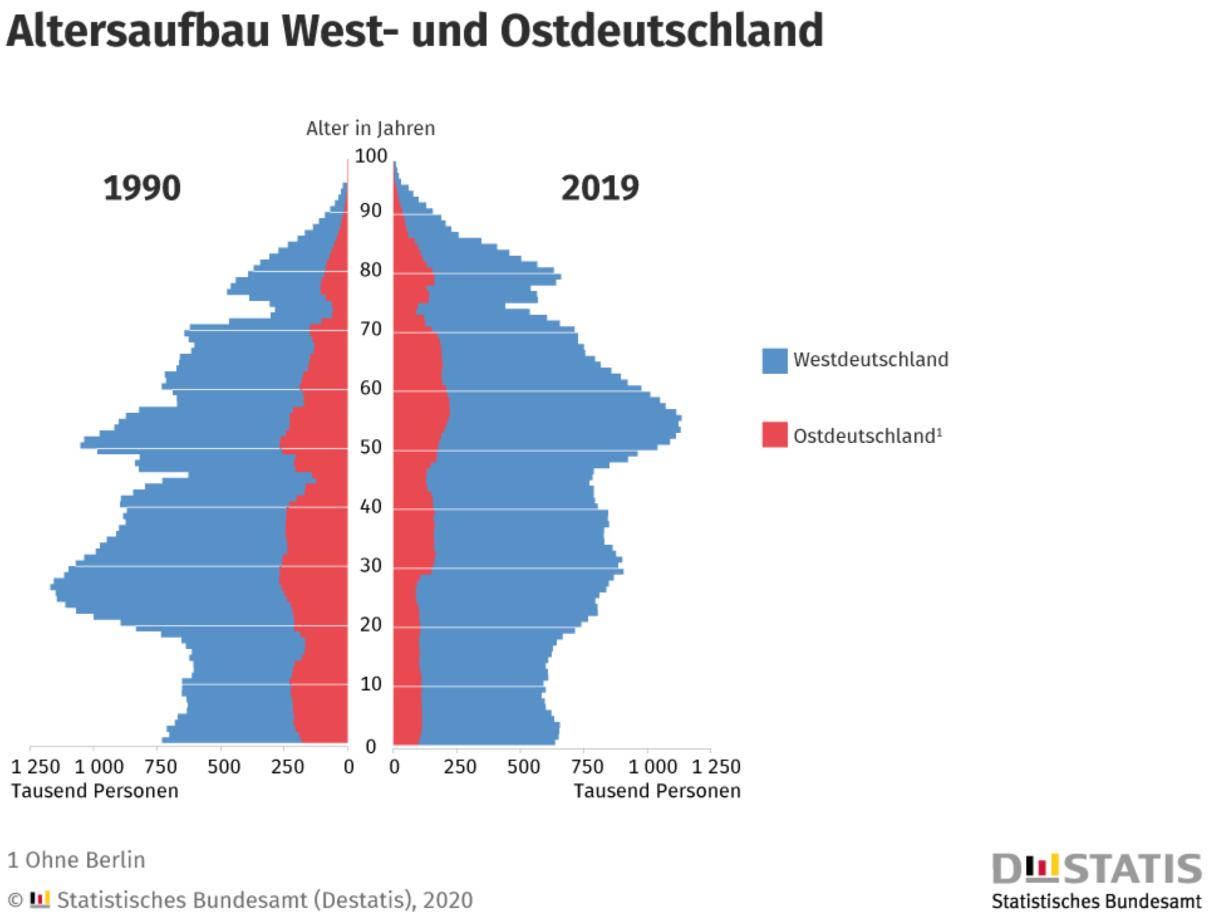

Figure S5: Age distributions of the population in West- (blue) and East Germany without Berlin (red) in 1990 and 2019. Graphic © Statistisches Bundesamt 2020, obtained in April 2021 via URL https://www.destatis.de/DE/Themen/Querschnitt/Demografischer-Wandel/_Grafik/_Statisch/demografischer-wandel-30-jahre-de-altersaufbau.png.

This can be explained by the age structure (Fig. S5): The fertility among the population remaining in this part of the country is unsustainably low for decades, at about one child per woman in the 1990s, now stabilizing at 1.6 children (DESTATIS 2019a). The recent immigration of mostly younger refugees hardly altered the picture: outside the big cities with up to 4 % refugee share regularly only 1.0–1.5 % of the population is backed up by refugees. Consequently there are now about two people in the age bracket of 50–70 years per child or young adult up to the age of 20 living in Eastern Germany, and Maretzke et al. (2021) expect an aged population in the rural parts still in the year 2040 with averages above 50 years.

S1.4 Economy

The economy in the GEB has undergone a process of deindustrialization since the German reunification. With the exceptions of the metropolitan regions of Hamburg, the international port city, and Berlin, the federal capital, the GEB comprises the poorest regions of Germany measured by average available income and personal net worth of the inhabitants. However, a 25 % share of the secondary sector in gross value added (GVA) and the general prosperity of the region are still comparable to EU averages. Table S2 summarizes the situation; the year 2019 was chosen for reference because 2020 data were affected by the onset of the global pandemic.

Table S2: National accounts and other economic key figures for the year 2019. Federal state codes: BE = Berlin, BB = Brandenburg, SN = Saxony, ST = Saxony-Anhalt, TH = Thuringia. GVA = Gross value added. NACE sector A = Agriculture, forestry, and fishing; NACE sectors B–F = Industry including mining, utilities, and construction; NACE sectors G-U = all other economic activities (the service sector). Sources: Statistische Ämter (2022), Eurostat (2022a).

Reporting item and unit		Federal states representing the GEB						For comparison	
		BE	BB	SN	ST	TH	Combined	Germany	EU 28
Gross domestic product	bn €	157.5	76.2	130.5	64.8	63.9	492.9	3 473.4	16 543.8
GDP per inhabitant	1000 €	43.1	30.3	32.0	29.4	29.9	33.8	41.5	32.2
Disposable income per inhab.	1000 €	21.4	21.7	21.1	20.5	20.6	21.1	23.6	n/a
GVA share NACE sector A	%	0.0	1.5	0.8	1.9	1.3	0.9	0.8	1.6
GVA share NACE sectors B–F	%	12.5	24.9	28.2	29.6	30.3	23.2	27.0	24.5
GVA share NACE sectors G–U	%	87.5	73.6	71.0	68.5	68.4	75.9	72.2	73.9

S2 Drought indices

S2.1 The Standardized Precipitation-Evapotranspiration Index (SPEI) in the Havel area

Figure S6 is the Havel area analog to the SPEI time series of the GEB shown in Fig. 2 of the main article. As already mentioned there, differences between the two figures are hard to spot.

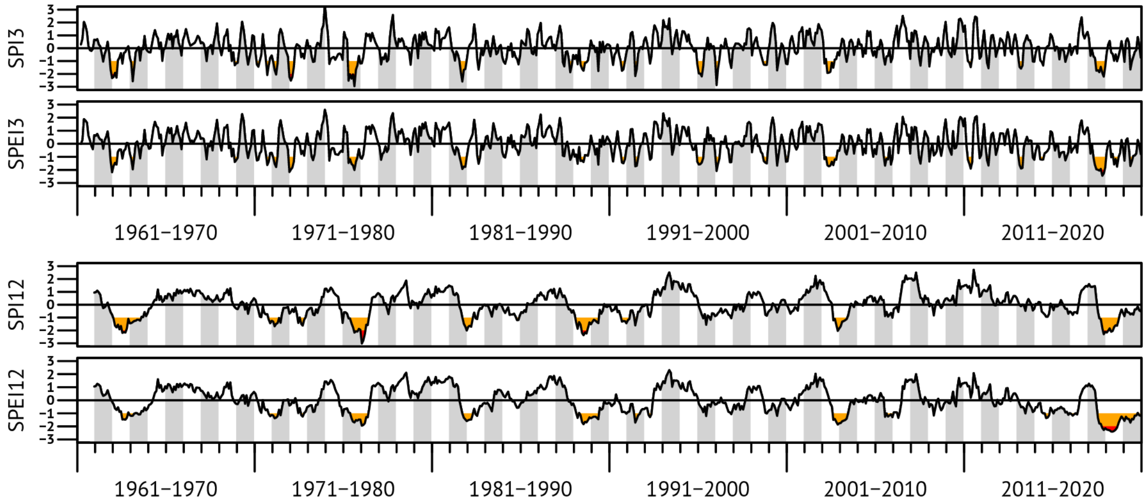

Figure S6: Meteorological drought indices for the Havel area, calculated for 3- and 12-month running averages. The orange and red threshold levels are at −1.0 and −2.0 indicating drought and extreme drought, respectively.

S2.2 Maps of soil drought from the UFZ Drought Monitor

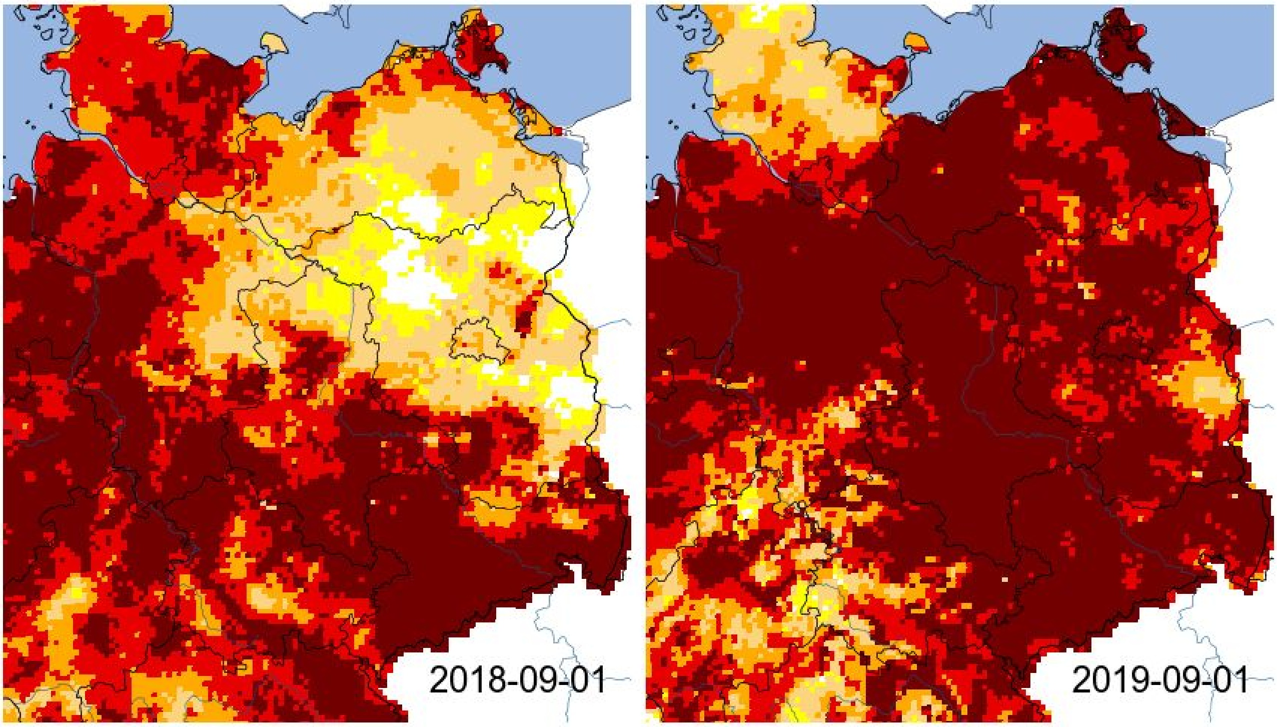

Figure S7: Spatial distribution of full profile (1.8 m) SMI extremes in Northeastern Germany after the summers of 2018 and 2019. Orange colours indicate drought (values of 0.2 or less); scarlet (0.05 or less) and brown red (0.02 or less) show extreme and exceptional drought, respectively. Source: UFZ Drought Monitor / Helmholtz Centre for Environmental Research.

Figures S7 and S8 give an impression of the soil drought persistence in Northeastern Germany. Correlation analyses between SPEI and SMI show the highest correlations for 18–24 month SPEI scales, the highest hysteresis observed within Germany.

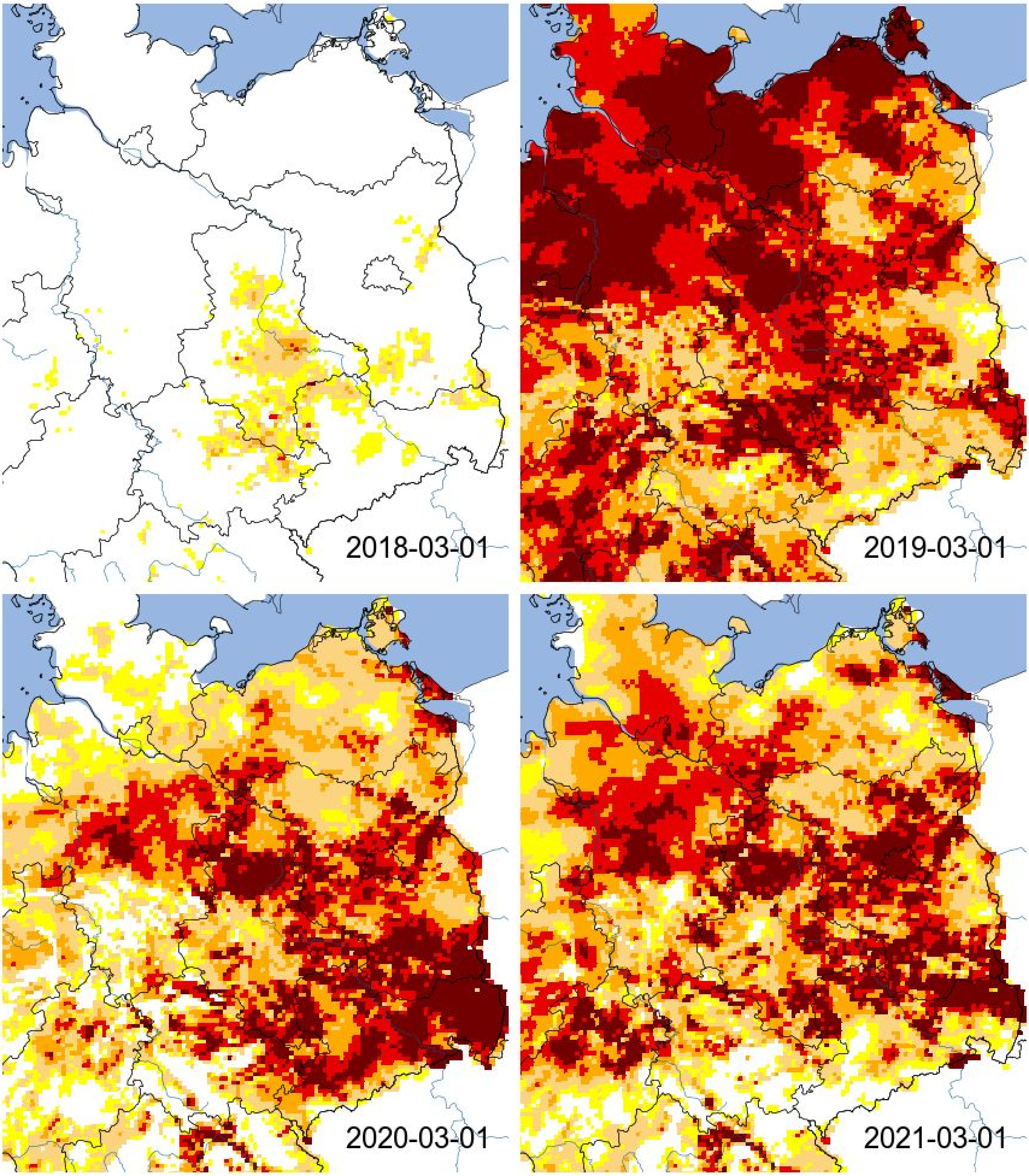

Figure S8: Spatial distribution of full profile (1.8 m) SMI extremes in Northeastern Germany at begin of spring for the years 2018–2021. Orange colours indicate drought (values of 0.2 or less); scarlet (0.05 or less) and brown red (0.02 or less) show extreme and exceptional drought, respectively. Source: UFZ Drought Monitor / Helmholtz Centre for Environmental Research.

S2.3 Calculation of the Standardized Streamflow Index (SSI)

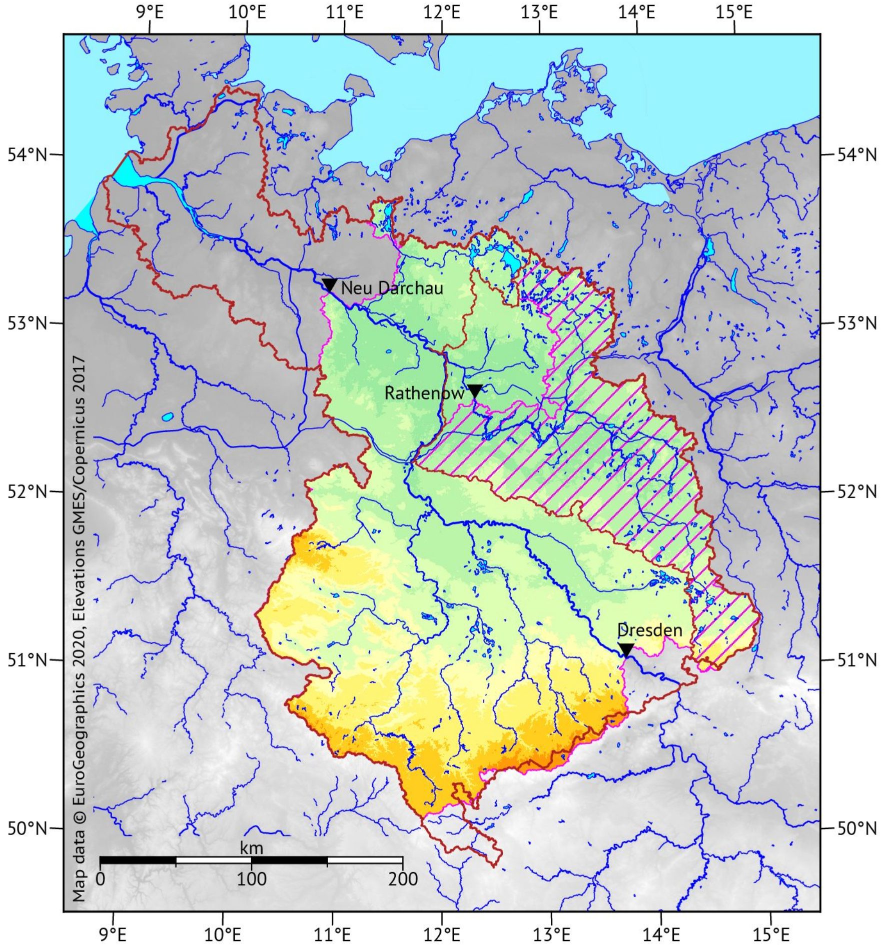

Figure S9: Stream runoff gauge catchments utilized for SSI computations. GEB is approximated by the Neu Darchau minus the Dresden catchment (area with elevation colours), and the Havel area is represented by the Rathenow catchment (hatched).

Like the meteorological drought indices SPI and SPEI the SSI compares monthly runoff values to distributions of other runoff records of the same month and provides the results in a normalized way as z-values. At the core of the Best Monthly Fit (BMF) method is the selection of a probability distribution from a candidate set to optimally approximate the data distribution for each calendar month separately. We used the candidate distributions proposed by Vicente-Serrano et al. (2012) (GEV, PearsonIII, Generalized Pareto, Lognormal, Log-Logistic, and Weibull), but did not fit the distribution parameters through L-moments but maximum likelihood estimation. The selection of the distribution to apply was based on *p*-values calculated by the Kolmogorov–Smirnov-test. Table S3 lists distributions and *p*-values for the catchment areas and calendar months.

Interestingly, the distribution of the monthly streamflows generated from the catchment area between the Elbe gauges representing the GEB (Fig. S9) could be approximated best by either lognormal or log-logistic probability distributions while the Havel streamflow at Rathenow required a bunch of different distributions. The *p*-Values are generally lower for the Havel catchment, probably due to noise in the Elbe data remaining from incomplete filtering of the inflow signal from upstream Dresden.

Table S3: Probability distributions selected in the Best Monthly Fit method of the SSI calculation and their p-Values according to KS-testing.

	Elbe basin (Dresden–Neu Darchau)		Havel catchment, gauge Rathenow	
Month	Prob. distribution	p-Value	Prob. distribution	p-Value
January	Log-Logistic	0.068	PearsonIII	0.077
February	Log-Logistic	0.111	Lognormal	0.070
March	Lognormal	0.085	Lognormal	0.106
April	Lognormal	0.064	PearsonIII	0.067
May	Log-Logistic	0.079	Gumbel	0.064
June	Log-Logistic	0.090	PearsonIII	0.053
July	Lognormal	0.055	Weibull	0.070
August	Lognormal	0.072	Lognormal	0.057
September	Lognormal	0.088	PearsonIII	0.074
October	Log-Logistic	0.070	Lognormal	0.043
November	Log-Logistic	0.111	PearsonIII	0.046
December	Log-Logistic	0.062	Weibull	0.056

S3 Aspects of the socio-economic drought impacts

S3.1 Agriculture

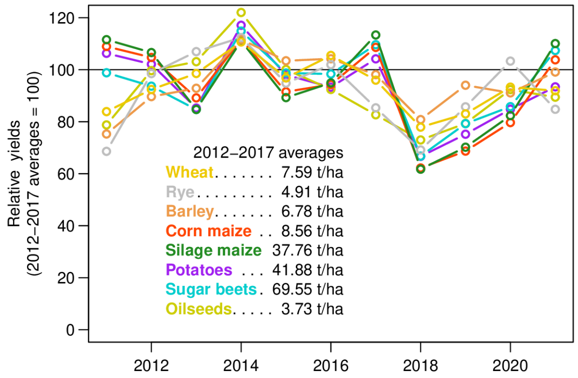

Figure S10: A decade of relative yield levels for eight crops in the GEB (approximated by the federal states Brandenburg, Saxony, Saxony-Anhalt, and Thuringia). The crop-specific values are normalized based on the 2012–2017 yield averages (y = 100) listed in the colour legend. Data source: DESTATIS (2012–2022).

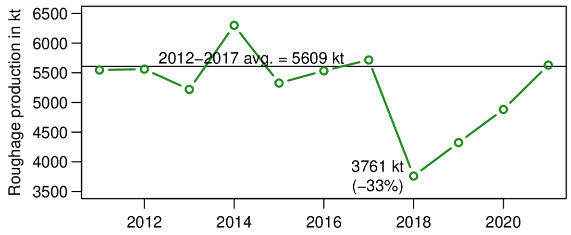

Figure S11: A decade of roughage harvests in the GEB (approximated by the four states as in Fig. S10)

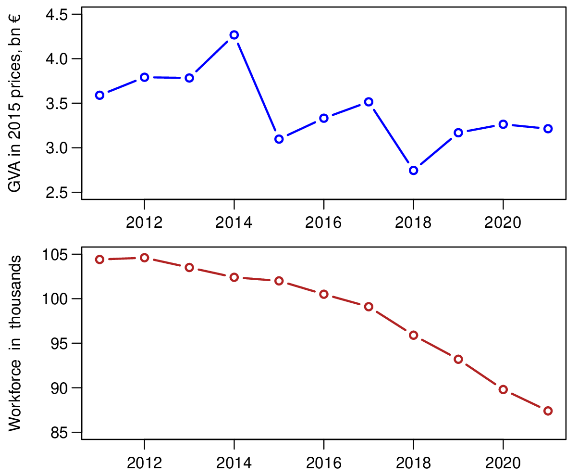

Figure S12: A decade of gross value added (GVA) and workforce in agriculture and (practically irrelevant) fisheries. Four-state approximation for the GEB. Data source: Statistische Ämter (2022).

While Figs S10 and S11 illustrate the seizable drought impact on plant production, the primary economic sector in general was affected more by background downtrends as shown in Fig. S12.

S3.2 Forestry

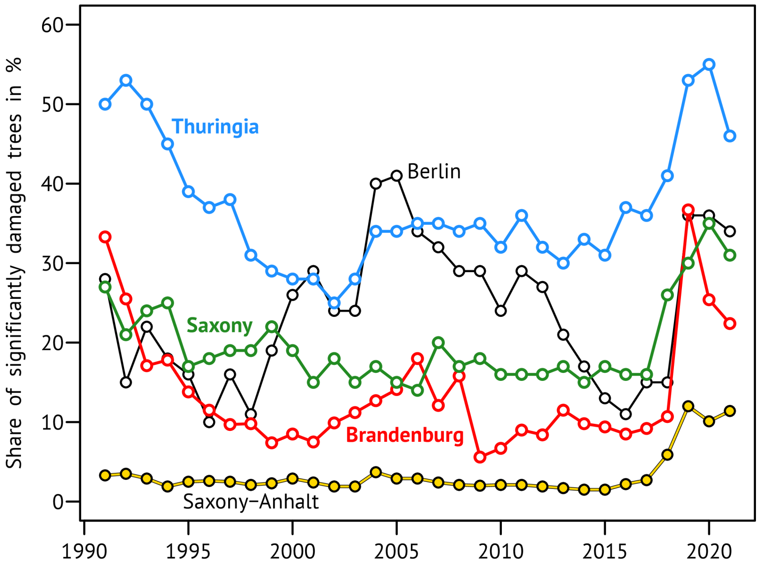

Figure S13: Percentages of forest trees with significant damages (stages 2–4) in the five federal states. For Saxony-Anhalt, only stages 3 and 4 (severe damages) are shown. Data sources: SVUVK (2021), MLUK (2021), SMEKUL (2021), MULE (2021), TMIL (2021); Methodology: Wellbrock et al. (2018).

Table S.4: Area affected by forest fires in the four federal states. Source: BLE (2016–2022)

Year	Burnt forest area in hectares					
	Berlin	Brandenburg	Saxony	Saxony-Anhalt	Thuringia	All five states
2015	0.0	328.3	16.5	31.4	6.4	382.6
2016	1.3	157.4	5.4	30.8	2.0	196.9
2017	0.4	285.4	4.9	5.1	1.2	297.0
2018	0.8	1674.1	305.5	113.9	11.2	2105.5
2019	13.9	1388.6	56.8	20.6	21.6	1501.5
2020	0.1	118.7	32.8	8.9	9.0	169.5
2021	0.0	33.9	3.2	22.6	1.7	61.4

S3.3 Inland fisheries

A report of the situation of the German inland fisheries and aquaculture is ordered annually by the supreme fishery authorities of the federal states. The issue for 2019 (Brämick n.d.) counted 166 professional fishing and 290 aquaculture enterprises for the five-state aggregate. Catches from rivers and lakes most of which are located in Brandenburg amount to approximately 1200 t per year, and the aquaculture production, centred in Saxony, reached about 4250 t/year. Using rough estimates of 15.00 €/kg and 7.50 €/kg as average retail prices for wild and aquaculture-produced fish, respectively, yields a gross revenue estimate of about 50 million euros.

Due to the direct consequence of the lack of water on fisheries, this sector is directly endangered by drought conditions. The case of the Seddin Lake (*Großer Seddiner See*; 52°16.5′N, 13°02.0′E; approx. 30 km south-west of Berlin) whose fisherman had to close shop due to low water levels was however more likely caused by sub-optimal water management than by drought alone.
With SN and BB producing 47 % of carp in Germany, carp farming is a traditional kind of aquaculture in the Elbe River basin (Edebohls et al. 2021). Carp ponds are shallow water bodies with water depths around one meter. The ponds are drained in an annual cycle in order to harvest the carps. The water bodies differ in terms of water provision. In SN 25 % of the ponds are fed only from precipitation in mostly small basins. Other ponds are fed by above ground streams or groundwater (Ballmann et al. 2017). Salmonids are produced in flowing water sites. They prefer cold water temperatures and high oxygen levels. The most produced salmonid in Germany is the rainbow trout.

Brämick (n.d.) reports increased fish mortality for 2018 and 2019 owing to high temperatures causing lack of oxygen, and dried-out ponds limiting the production. In this case the fishes are usually harvested before complete losses would be inevitable. However, carps are a warmwater species tolerating low oxygen levels (Ballmann et al. 2017). Therefore sites which held a minimum amount of water and level of oxygen had low economic losses.

The production volume of carp in Saxony stayed relatively stable during the drought years 2018–2020. With the minimum of 1674 tonnes of carp reached in 2017 the production was between 5 % and 7 % lower than the average value of the years 2012–2017. The number of businesses producing carp in SN decreased about 20 % from an average of 156 in the years 2015–2017 to 125 in 2020. The loss for rainbow trout producers were higher. The production of the temperature sensitive fish declined by 39 % compared to the the 2012–2017 average (DESTATIS 2012–2021).

In Brandenburg the relative loss in carp and rainbow trout production was higher than in SN. The production declined by 19 % in 2018 and 26% in 2019 for carp and 34% (2018) and 33% (2019) for rainbow trout compared to the averages of the years 2013–2017 (Statistik BB 2016–2020, DESTATIS 2012–2021). In TH the average production of trout from aquacultures diminished by 19 % during the drought years 2018 and 2019 compared to the 2012–2017 period. Carp production was 13 % below the 2012–2017 average (DESTATIS 2012–2021).

S3.4 Manufacturing

Table S5: Especially water or energy sensitive sectors of manufacturing (Auerswald & Vogt 2010)

Economical (sub-)section	Energy sensitive	Water sensitive
Food and tobacco processing		X
Paper, publishing, and print		X
Chemicals and chemical products	X	X
Rubber and plastic products	X	
Non-metallic mineral products (glass, ceramics, etc.)	X	X
Metals and metal products (except machinery)	X	

S3.5 Mining and related water issues in the GEB

The millennial history of mining in the mountainous areas, mainly for non-ferrous and noble metals, after WWII also for uranium in the Ore mountains, ended with the 20th century. Still in operation are only a few open-cast mining areas for lignite, remains of two furthering regions (*Mitteldeutsches Revier, Lausitzer Revier*) that reached their production peak already in the 1980s. and are now characterized by remodelled landscapes: disused mining pits usually being transformed into lakes.

Water was practically always more a disturbance than an advantage for mining activities (with the exception of early mechanical hydropower applications, e.g. for hammer mills). The open-cast lignite mining requires constant groundwater withdrawals through electric pumps to keep the excavation areas dry. Consequently, the recent drought was no problem at all for mining as such.

Before the German Reunification in 1990 up to 30 m³/s of groundwater were extracted from the Lusatian mining area and released into the Spree river. Meanwhile, most former open-cast mining areas are being flooded and groundwater storage recovers which naturally means less streamflow contribution (Grünewald 2001, 2010; Koch et al. 2005). Consequently the 1976 and 1989 droughts are less pronounced in the Havel SSI compared to the time series for the entire contribution area between the Elbe gauges, while the downward SSI peak of 2018 was more negative for the Havel subbasin. Other upstream areas of the GEB may expose streamflow modifications from reservoir operations, the reservoirs in the Harz and Ore mountains are however small and can neither alter the general runoff seasonality nor provide sustained runoff during drought.

The planned emergence of lakes and waterways in the former mining area landscapes was however hampered by the drought. A water management centre in Lusatia, the border region of Brandenburg and Saxony, has been installed to carefully balance the filling of the new lakes with the minimum runoff requirements of the Spree River crossing Berlin. Shifting the goal of fully restored groundwater storages and filled artificial lakes (currently the year 2090 is aimed for the final lake fill; Scholz & Totsche 2022) further into the future is less problematic than a stagnant Spree river with water quality issues amidst the German capital (which still could be avoided).

S3.6 Energy and Water supplies

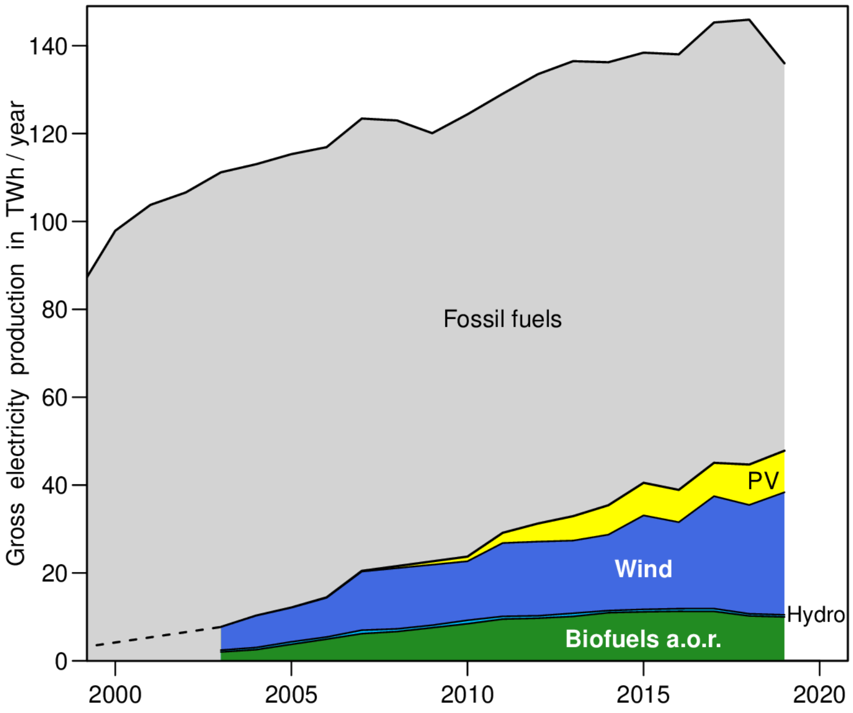

Figure S14: Gross electricity production in the five-state aggregate separated by contributions of fossil fuels and renewable sources. Data source: LAK Energiebilanzen (2022).

A negative effect of the 2018 drought on the energy supply can be spotted in the biofuel share (green band in Fig. S14): After several years of relatively stable contributions it decreased from 10.9 TWh in 2017 to 10.0 TWh in 2018 – obviously crop failures led to fuel limitations. It is also traceable in the electricity output of Berlin's hardcoal plants: only 2.8 TWh in 2018 after 3.7, 3.5, and 3.4 TWh in the three previous years; these plants had to be throttled due to limitations in cooling water. The share of these Berlin plants in the fossil fuel based electricity production of the five-state aggregate (grey band in Fig. S14) is however negligible.

The drought had a positive effect on the five-state figures for photovoltaics (PV, yellow band in Fig. X.23). The output jumped to 9.2 TWh in 2018 after 7.4, 7.4, and 7.6 TWh in the three previous years. Hydropower shows of course the opposite, but it is of no practical importance despite the mountainous areas of the GEB being fully covered by the five states: 482 GWh in 2018 after 545, 579, and 616 GWh in the years 2015–2018 (LAK Energiebilanzen 2022).

There is a non-free database of the Energy sector collecting member reports on power production reductions and the reasons for them (KISSY, VGB Power Tech e.V.). The most recent free excerpt shows temperature-related production shortages of thermal power plants on the national level (IMAA 2019), but does not contain the years after 2017. Until then, the highest shortages were in 2003 with 2500 GWh and in 2006 with 1775 GWh. Power plants along the Elbe river had however no share in the 2003 reductions which concentrated on nuclear plants in Western Germany. For 2018, there are a number of press reports about throttled power generation in Germany, but the two nuclear plants on the lower part of the Elbe River, Brunsbüttel and Stade, had already been shut down before.

While the recent drought did not cause any relevant outages in industrial and drinking water supply, record low groundwater levels and the perspective of decreasing water balances and groundwater recharge under climate change pose the question whether the high reliability of the system can still be guaranteed in the future. The associations of engineers, utility companies and water suppliers (the latter usually being public corporations in Germany) did their homework analysing the situation (Simon et al. 2019, DVGW 2021) and drafted a demand charter (BDEW-DVGW-VKU 2021). The German association of cities and municipalities took the same line (DStGB 2022), and the federal government published the draft of a national water strategy (BMU 2021).

These proposals build on the compound power of numerous measures drawn from the engineer's space of possible solutions. Tap water security is definitely given a high priority, and large sums of public money and water fees will be invested over time, for instance in new wells, additional pipe connections, forest conversions, rainwater processing plants, or intelligent demand monitoring and charging systems. In the long term, the price of tap water (and probably also taxes) will consequently rise a lot, and that will be the economic drought burden – not directly connected to any particular drought event.

Tap water fees change between supply areas, often municipalities. In 2019, an average German household paid approximately € 242 per year for their water (DESTATIS 2020), state averages were highest in the Saarland (€ 310) and North-Rhine Westphalia (€ 298). Within the Elbe area, tap water was most expensive in Thuringia (€ 267) followed by Hamburg (€ 256) and Saxony (€ 246). Interestingly, the average annual household fee is only € 157 in Berlin where the per-capita water availability is obviously most limited.

In addition to these water fees private end-users have to pay for the sewage water removal, usually a similar amount, so the average total water fees are currently about 400–500 € per household and year. This may probably rise by 20–30 % in the 2020s to finance the investments necessary for continued supply security. A surplus water fee of 125 € per year and household could grant about 5 billion € annually for infrastructure investments (1 billion € per year for the GEB), but any discussion about utility costs is currently focused on energy, and it is impossible to give sound price projections in times of international tensions and generally accelerating inflation.

S3.7 Construction

No hard data were available about drought effects on construction productivity, therefore this segment is not explicitely mentioned in the main article. The freshwater use of the construction sector is relatively low, with 44 million m³ in 2016 only at 1.4 % of the private household demand of 3118 million m³ (DESTATIS 2019b), and with practically no tap water restrictions there were no water shortage problems.

Weather is nevertheless a bigger issue in construction; some outdoor works cannot be done in bad weather conditions, but sunny days with strong radiation and high temperatures limit the human productivity outdoors. A global study (ILO 2019) found construction among the most affected branches but quantified the climate-change related productivity losses in Western and Eastern Europe in small fractions of a percent; losses during hot summer days may be balanced by shorter bad weather breaks in winter (IMAA 2019).

A special factor to take into account for construction workers is the additional heat stress through the protective clothing. Other stresses, namely increased ozone levels and uv radiation, need also to be taken into account. For construction workers in Germany skin cancer is a recognised occupational disease.

One of the most comprehensive summaries about heat effects on outdoor workers has been published in the online magazine of the statutory accident insurance institution (*Berufsgenossenschaft*) for the German construction sector (Pohrt et al. 2021).
There are however non-monetary losses: Accidents and fatalities become more frequent under high temperatures. This was tragically demonstrated by the death toll for the construction of football stadiums for the 2022 FIFA world cup in Qatar, but the effect is also statistically evident in the United States (Pohrt et al. 2021) and thus very probably a neglected problem in Germany, too.

S3.8 Transport

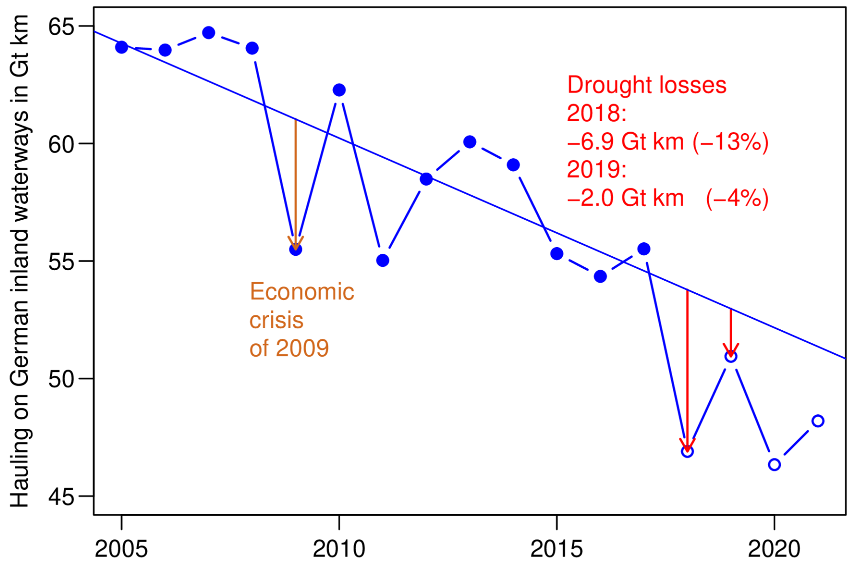

Figure S15: General downtrend in German inland navigation with special setbacks in single years. The trend line has been fitted to the pre-drought/pre-pandemic data of 2005–2017 (filled circles). Data source: DESTATIS (2022a).

Unfortunately, the statistics about hauling on inland waterways are inconsistent with respect to units. Most objective are ton-kilometres (load times distance), but these numbers are not easily available for subregions of Germany (monthly values are scattered about monthly reports). We therefore show the ton-kilometres for all of Germany in Fig. S15 and load-based indices for the relevant reaches of the Elbe River in Fig. S16. Table S5 illustrates the relative irrelevance of inland navigation upstream the port of Hamburg.

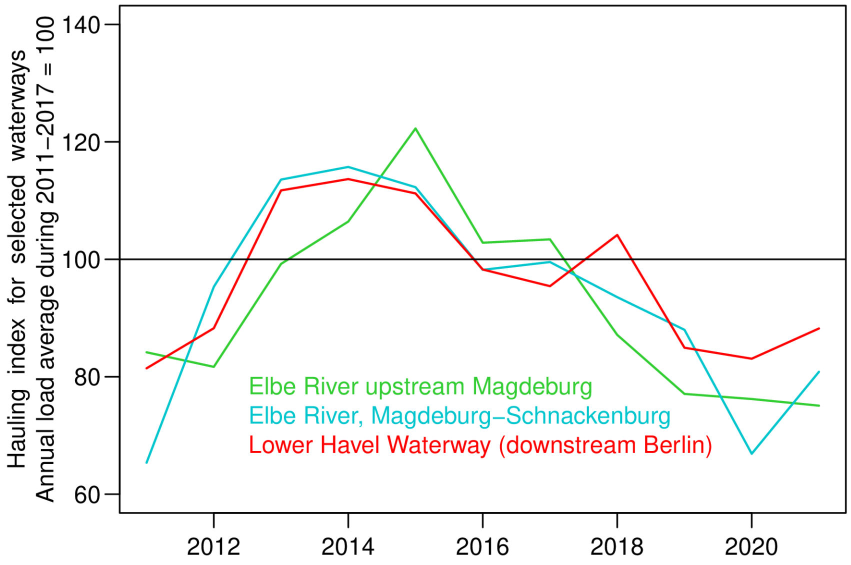

Figure S16: Recent hauling trends on three principal navigable waterways in the German Elbe basin. Index values of 100 represent the average annual load of the years 2011–2017. The respective absolute values were 3.673 million tons for the Elbe River upstream Magdeburg, 1.142 million tons for the Elbe reach Magdeburg–Schnackenburg, and 3.729 million tons for the Lower Havel Waterway. Data source: DESTATIS (2022a).

Table S6: Cargo loads transported in 2017 on GEB inland waterways in comparison to other routes of navigation within Germany. Data source: DESTATIS (2022a) unless noted otherwise

Cargo loads transported on major inland waterways of the GEB	
Elbe upstream Magdeburg	3 797 413 t
Elbe, Magdeburg–Schnackenburg	1 136 464 t
Lower Havel Waterway (Berlin–Elbe River)	3 434 159 t
For comparison: Cargo loads transported on German sections of the River Rhine	
Rhine, Mannheim–Bingen (includes Main River junction towards Frankfurt)	59 258 267 t
Rhine, Lülsdorf–Orsoy (passes cities of Cologne, Düsseldorf, and Duisburg)	148 278 673 t
For comparison: Offshore cargo handling through the Elbe River estuary	
Port of Hamburg	118 760 726 t
Port of Hamburg, according to Port of Hamburg (2022)	136 400 000 t

S3.9 Heat-wave related peaks in air conditioning demand

The market for air conditioning systems can serve as indicator for uncomfortable heat in offices and private homes. There is a highly detailed statistics about production and trade for EU countries maintained by Eurostat: PRODCOM (Eurostat 2021b). The relevant product group has got the PRODCOM code 28.25.12.20 – "Window or wall air conditioning systems, self-contained or split-systems". Figure S17 shows Germany's international trade in this kind of air conditioning.

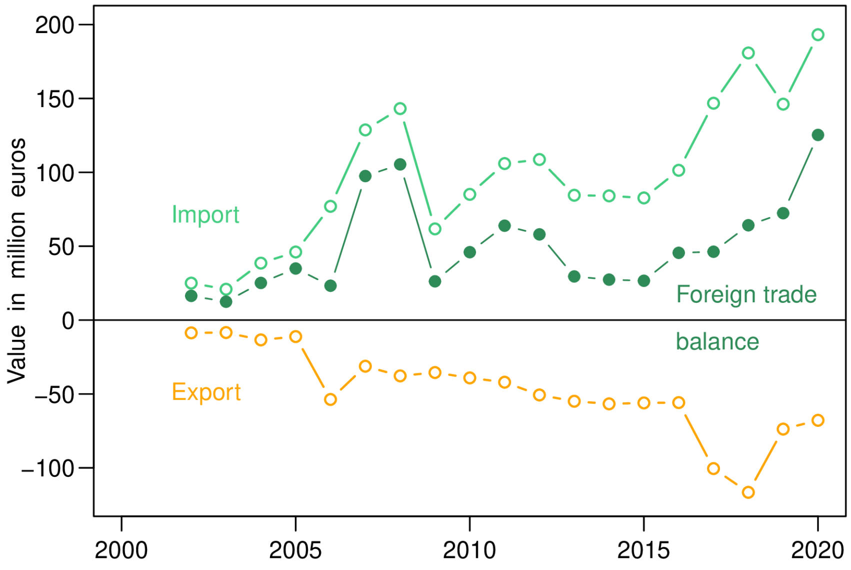

Figure S17: Foreign trade of professional window or wall air conditioning systems (Data source: Eurostat 2022b)

In contrast to production figures, the import/export values have a high coherence and reliability, because German customs scrutinize every parcel crossing the border. The net balance of the foreign trade reveals a clear but hysteretic response to the 2006 heat wave: In 2007 and 2008 the imports exceeded the exports by about 100 million euro p.a. The same hysteresis can be observed for the recent drought: It took until 2020, two years after drought onset, to return to a clearly elevated level of annual net imports.

Aside from that, it seems that the production of air conditioning sets in Germany gained momentum in the first years of the millenium, and there might be still an increasing trend in output while the average prices fell from about 500 €/unit ten years ago to about 300 €/unit recently. Hence air condition will probably become less extraordinary in the GEB in the near future.

S3.10 Heat-related performance losses in the service sector

The economic effect of heat-related performance losses in office work is hardly quantifiable. Zhao et al. (2021) reviewed 30 scientific articles on heat-related labour productivity losses and identified four different methods to quantify the economic impacts. The results from different world regions varied significantly and are not transferrable to the Elbe region. A similar meta-analysis of 35 studies was provided by Porras-Salazar et al. (2021) and came up with the shattering conclusion: “We could not find a relationship between temperature and office work performance neither for the range of temperatures measured in most of the office buildings (20°C–30°C) or a wider range (18°C–34°C). [...] The lack of relationships does not necessarily refute that temperature affects the performance of office work.” While the latter statement seems self-evident, both meta studies show that a generally applicable formula for the relationship between temperature and productivity of office workers is not on the horizon yet.

These findings question the validity of any single study such as IMAA (2019) estimating 540 million to 2.4 billion euro heat stress-related productivity losses for the entire German economy. And indeed: IMAA (2019) cite Hübler & Klepper (2007) as source for the 3–12 % loss range their figures

are based on, who in turn give Bux (2006) as source. However, Bux (2006) also discusses inconsistent findings in the literature and mentions the 3–12 % range without context, just as example for the typical uncertainty!

The German weather service (DWD) uses the “Klima-Michel” model for the thermal comfort of a reference person walking *outdoors* with moderate speed (Jendritzky et al. 1979, Jendritzky 1990). It is based on heat balance calculations for the human body and considers not only air temperature but also humidity, wind speed and radiation. A “perceived temperature” was developed as related index; values above 20 degrees indicate heat stress, different intensity levels are defined (Staiger et al. 1997). New insights about biometeorology and the temperature regulation of the human body led to the development of an overhauled index, UTCI (Jendritzky et al. 2010, http://www.utci.org).

A comparably refined thermal comfort index for *indoor* conditions would probably be needed to reliably describe and parameterize the relationship between thermal environment and human productivity in offices. Air humidity is still a neglected factor in productivity research, but also air movements from ventilation and heat radiation sources may be influential for office environments. Furthermore, different tasks are affected differently by heat stress (Lan et al. 2009), hence we have to conclude that no reliable forward estimation of productivity losses under heat waves is possible yet.

S3.11 Defence

The only military stationed in Eastern Germany, and consequently in most of the GEB, is the German army (*Bundeswehr*), other NATO allies have to stay outside this region as stipulated in Article 5 of the reunification treaty, officially “Treaty on the Final Settlement with respect to Germany” (BGBl. 1990 II S. 1317). The Ministry of Defence in Berlin and the military headquarters near Potsdam are also located in the GEB. How the defence readiness condition is affected by drought belongs of course to the domain of state secrets, but there were some news regarding military operations and forest fires:

In 2018, the army supported the fighting of a wildfire at Treuenbrietzen, about 80 km south-west of Berlin, with helicopters and tanks. Also in 2018, a fire broke out in Saxony during a maneuver but was successfully extinguished by the military fire fighters at the same day. Another wildfire burning more than 900 ha of forest started in 2019 on the former military training range Lübtheen (about 53°17’N, 11°09’E). Due to the site’s contamination with ammunition (it had been used for shooting continuously during 1936–2013) army troops and regular firefighters could not operate from a short distance.

S3.12 Education

Schools responded to the extreme heat events during summer months of 2018 and 2019 with shortened lessons or cancellation of courses. In BB and ST changes in the timetables are regulated by temperature measurements in classrooms or on the schoolyard, whereas in SN and in TH respective decisions are made independent from pre-defined thresholds.

S3.13 Human health: UV radiation

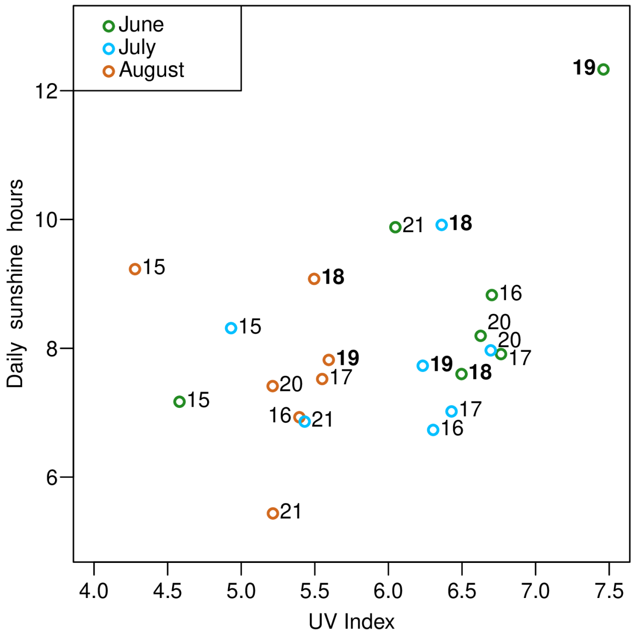

Figure S18: UV index and daily sunshine hours in the summer months of the years 2015–2021 observed at Lindenberg (52°12’32″N, 14°07’03″E). The two-digit numbers refer to the calender years, months are colour-coded.

Fig. S18 is referenced in Section 3.3 of the main article. Note that both UV indices and sunshine hours of the years 2018 and 2019 (highlighted by bold numbers) are tendentially above average, but notwithstanding the June 2019 record they are not exceptionally high.

S3.14 Arts, entertainment and recreation

The extreme drought conditions increased the potential of wildfires during 2018 and 2019 and thereby forced music and art festivals to delay or cancellation. Traditional Easter fires were cancelled for the same reason in 2019. As a measure of fire prevention, entering and visiting forest areas at night was not allowed in the Saxon national park “Sächsische Schweiz” during spring and summer of 2019.

The drought events affected tourism in the GEB in multiple ways. Forms of tourism depending on water availability in the first order were most affected. For instance, boating trips could not take place on the Elbe River during the 2019 drought. In 2020 the drought had impacts on canoeing tourism: locks had to be kept closed in order to maintain water levels.

Camping tourism is generally furthered by sunny weather conditions. Statistics of camping tourism in the five federal states (DESTATIS 2022b, amended by Statistik BB 2017–2019 and Statistik ST 2019) agree in increasing numbers of arriving visitors and overnight stays from the year 2018 on. Compared to the three-year averages of 2015–2017 (924 707 arrivals and 2 804 310 overnight stays) the increases in arriving visitors amounted to 21 % in 2018, to 31 % in 2019, and to 33 % in 2020. Overnight stays increased by 17 %, 27 %, and 35% respectively.

The statistics also contain the numbers of foreign visitors to camping sites, but only SN and TH for each of the years 2015–2020. In Saxony, the share of overnight stays spent by foreign visitors decreased from 13 % in 2015 to 11 % in 2019, and then dropped to less than 4 % in 2020. In Thuringia, a stable foreigner share of 7 % was observed in the years 2015–2017; this decreased to 6 % in 2019, and finally dropped to 3 % in 2020. The increased share of local tourists at campsites during 2018 and 2019 suggests more spontaneous camping trips attracted by the sunny weather conditions. The disappearance of foreign visitors in 2020 was however a result of legal travel restrictions motivated by the pandemic situation.

Swimming pool operators clearly benefitted from the warm and dry swimming seasons; several visitor records have been thrown during the warm summer months of 2018.

References

Auerswald H, Vogt G (2010) Zur Klimasensibilität der Wirtschaft in der Region Dresden. ifo Dresden berichtet 17(3):15–23. https://www.ifo.de/DocDL/ifodb_2010_3_15_23-3.pdf – Last accessed in July 2022.

Ballmann H, Bärisch S, Böhm A, Franke J, Füllner G et al. (2017) Auswirkungen des Klimawandels auf die Perspektiven in der sächsischen Teichwirtschaft. Landesamt für Umwelt, Landwirtschaft und Geologie (LfULG), Dresden, und Staatsbetrieb Sachsenforst (SBS), Graupa, 42 pp. https://slub.qucosa.de/api/qucosa%3A16119/attachment/ATT-0/ – Last accessed in July 2022.

BDEW-DVGW-VKU (2021) Bedarfe der Wasserversorgung in Zeiten des Klimawandels. Maßnahmenvorschläge des BDEW, DVGW und VKU zur Sicherung der Wasserversorgung. DVGW Deutscher Verein des Gas- und Wasserfaches e.V., Bonn; BDEW Bundesverband der Energie- und Wasserwirtschaft e.V., Berlin; VKU Verband kommunaler Unternehmen e.V., Berlin; 4 pp. https://www.dvgw.de/medien/dvgw/verein/aktuelles/presse/BDEW_DVGW_VKU_Positionspapier_Klimawandel_Trockenheit.pdf – Last accessed in July 2022.

BKG (2021) Verwaltungsgebiete 1:250 000 mit Einwohnerzahlen (Ebenen), Stand 31.12. (VG250-EW). Digital vector maps. Bundesamt für Kartographie und Geodäsie, Frankfurt am Main. https://gdz.bkg.bund.de/index.php/default/open-data/verwaltungsgebiete-1-250-000-mit-einwohnerzahlen-ebenen-stand-31-12-vg250-ew-ebenen-31-12.html – Last accessed in July 2022.

Błażejczyk K, Jendritzky G, Bröde P, Fiala D, Havenith G, Epstein Y, Psikuta A, Kampmann B (2013) An introduction to the Universal Thermal Climate Index (UTCI). Geographica Polonica 86(1):5–10. doi:10.7163/GPol.2013.1

BLE (2016–2022) Waldbrandstatistik für die Bundesrepublik Deutschland [annual issues, published online as PDF documents]. Bundesanstalt für Landwirtschaft und Ernährung, Bonn. https://www.bmel-statistik.de/forst-holz/waldbrandstatistik/ – Last accessed in August 2022.

BMU (2021) Nationale Wasserstrategie – Entwurf des Bundesumweltministeriums. Bundesministerium für Umwelt, Naturschutz und nukleare Sicherheit, Bonn, 76 pp. https://www.bmuv.de/fileadmin/Daten_BMU/Download_PDF/Binnengewaesser/langfassung_wasserstrategie_bf.pdf – Last accessed in July 2022.

Brämick U (no date) Jahresbericht zur Deutschen Binnenfischerei und Binnenaquakultur [annual issues, published online as PDF documents]. Institut für Binnenfischerei e.V., Potsdam-Sacrow. https://www.bmel-statistik.de/ernaehrung-fischerei/fischerei/aquakultur – Last accessed in July 2022.

Bux K (2006) Klima am Arbeitsplatz: Stand arbeitswissenschaftlicher Erkenntnisse – Bedarfsanalyse für weitere Forschungen. Projekt F 1987. Bundesanstalt für Arbeitsschutz und Arbeitsmedizin; Dortmund, Berlin, Dresden; 33 pp. https://www.baua.de/DE/Angebote/Publikationen/Berichte/Gd45.pdf – Last accessed in July 2022.

Cherlet M, Hutchinson C, Reynolds J, Hill J, Sommer S, von Maltitz G (eds) (2018) World Atlas of Desertification – Rethinking land degradation and sustainable land management. 3rd edn. Publication Office of the European Union, Luxemburg, 248 pp. ISBN 978-92-79-75350-3. doi:10.2760/06292

Copernicus (2020) CORINE Land Cover (CLC) 2018, version v.2020_20u1, downloaded in March 2021. Source: Copernicus Land Monitoring Service, part of the European Earth monitoring programme (GMES). URL: https://land.copernicus.eu/pan-european/corine-land-cover/clc2018

DESTATIS (2012–2021) Erzeugung in Aquakulturbetrieben. Fachserie 3 Land- und Forstwirtschaft, Fischerei, Reihe 4.6. Annual issues. Statistisches Bundesamt, Wiesbaden. https://www.statistischebibliothek.de/mir/receive/DESerie_mods_00000531 – Last accessed in July 2022.

DESTATIS (2012–2022) Wachstum und Ernte – Feldfrüchte. Fachserie 3 Land- und Forstwirtschaft, Fischerei, Reihe 3.2.1. Annual issues. Statistisches Bundesamt, Wiesbaden. https://www.statistischebibliothek.de/mir/receive/DESerie_mods_00000335 – Last accessed in July 2022.

DESTATIS (2019a) Bevölkerung im Wandel – Annahmen und Ergebnisse der 14. koordinierten Bevölkerungsvorausberechnung. Statistisches Bundesamt, Wiesbaden, 69 pp. https://www.destatis.de/DE/Presse/Pressekonferenzen/2019/Bevoelkerung/pressebroschuere-bevoelkerung.pdf – Last accessed in July 2022.

DESTATIS (2019b) Umweltnutzung und Wirtschaft – Tabellen zu den Umweltökonomischen Gesamtrechnungen. Teil 4: Wassereinsatz, Abwasser. Ausgabe 2019. Statistisches Bundesamt, Wiesbaden, 30 pp. https://www.destatis.de/DE/Themen/Gesellschaft-Umwelt/Umwelt/UGR/rohstoffe-materialfluesse-wasser/Publikationen/Downloads/umweltnutzung-und-wirtschaft-tabelle-5850007197006-teil-4.pdf – Last accessed in July 2022.

DESTATIS (2020) Entgelt für die Trinkwasserversorgung in Tarifgebieten nach Tariftypen 2017 bis 2019, Stand 26. Juni 2020. Online Table. Statistisches Bundesamt, Wiesbaden. https://www.destatis.de/DE/Themen/Gesellschaft-Umwelt/Umwelt/Wasserwirtschaft/Tabellen/tw-07-entgelt-trinkwasserversorgung-tarifgeb-nach-tariftypen-2017-2019-land-bund.html – Last accessed in July 2022.

DESTATIS (2021) Verwaltungsgliederung am 31.12.2020. Excel file, 46 kB. Statistisches Bundesamt, Wiesbaden. https://www.destatis.de/DE/Themen/Laender-Regionen/Regionales/Gemeindeverzeichnis/Administrativ/Archiv/Verwaltungsgliederung/31122020_Jahr.html – Last accessed in January 2022.

DESTATIS (2022a) Güterverkehrsstatistik der Binnenschifffahrt. Genesis-Online data base, Code 46321. Statistisches Bundesamt, Wiesbaden. https://www-genesis.destatis.de/genesis/online?operation=statistic&code=46321#abreadcrumb – Last accessed in July 2022.

DESTATIS (2022b) Ankünfte und Übernachtungen auf Campingplätzen (Urlaubscamping): Bundesländer, Jahre, Wohnsitz der Gäste. Genesis-Online data base, Table 45412-0023. Statistisches Bundesamt, Wiesbaden. https://www-genesis.destatis.de/genesis//online?operation=table&code=45412-0023#abreadcrumb – Last accessed in July 2022.

DStGB (2022) Wassermanagement in Zeiten von Hitze & Dürre. 10 Thesen & Forderungen des DStGB, Stand Mai 2022. Deutscher Städte- und Gemeindebund, Berlin, 2 pp. https://www.dstgb.de/publikationen/positionspapiere/aktives-wassermanagement-erforderlich/hitze-duerre-270522.pdf?cid=o2a – Last accessed in July 2022.

DVGW (2021) Wasserforschung zur Anpassung an den Klimawandel. Beiträge aus der DVGW energie | wasser-praxis. DVGW Deutscher Verein des Gas- und Wasserfaches e.V., Bonn, 66 pp. https://www.energie-wasser-praxis.de/fileadmin/heftarchiv/2021/ewp-dossier_wasserforschung.pdf – Last accessed in July 2022.

DWD-CDC (2022a) DWD Climate Data Center (CDC): Grids of monthly averaged daily air temperature (2m) over Germany, version v1.0. ASCII files. Deutscher Wetterdienst, Wiesbaden. URL: https://opendata.dwd.de/climate_environment/CDC/grids_germany/monthly/air_temperature_mean/ – last accessed in July 2022.

DWD-CDC (2022b) DWD Climate Data Center (CDC): Grids of monthly total precipitation over Germany, version v1.0. ASCII files. Deutscher Wetterdienst, Wiesbaden. URL: https://opendata.dwd.de/climate_environment/CDC/grids_germany/monthly/precipitation/ – last accessed in July 2022.

DWD-CDC (2022c) DWD Climate Data Center (CDC): Grids of monthly total sunshine duration over Germany, version v1.0. ASCII files. Deutscher Wetterdienst, Wiesbaden. https://opendata.dwd.de/climate_environment/CDC/grids_germany/monthly/sunshine_duration/ – Last accessed in July 2022.

DWD-CDC (2022d) DWD Climate Data Center (CDC): Monthly grids of the accumulated potential evapotranspiration over grass, version 0.x. ASCII files. Deutscher Wetterdienst, Wiesbaden. https://opendata.dwd.de/climate_environment/CDC/grids_germany/monthly/evapo_p/ – Last accessed in July 2022.

Edebohls I, Lasner T, Focken U, Kreiß C, Reiser S (2021) Steckbriefe zur Tierhaltung in Deutschland: Aquakultur. Thünen-Institute für Seefischerei und Fischereiökologie, Bremerhaven, 24 pp. https://literatur.thuenen.de/digbib_extern/dn063531.pdf – Last accessed in July 2022.

Eurostat (2022a) Eurostat data base (as of July 2022). https://ec.europa.eu/eurostat/data/database – Last accessed in July 2022.

Eurostat (2022b) Eurostat PRODCOM database: Sold production, exports and imports by PRODCOM list (NACE Rev. 2) - annual data, Last update 18-03-2022. https://appsso.eurostat.ec.europa.eu/nui/submitViewTableAction.do – Last accessed in July 2022.

FGG Elbe (2021) Zweite Aktualisierung des Bewirtschaftungsplans nach § 83 WHG bzw. Artikel 13 der Richtlinie 2000/60/EG für den deutschen Teil der Flussgebietseinheit Elbe für den Zeitraum von 2022 bis 2027. Textteil. Flussgebietsgemeinschaft Elbe, Magdeburg, 338 pp. https://www.fgg-elbe.de/berichte/aktualisierung-nach-art-13-2021.html – Last accessed in June 2022.

Grünewald U (2001) Water resources management in river catchments influenced by lignite mining. Ecol Eng 17(2–3):143–152. doi:10.1016/S0925-8574(00)00154-3

Grünewald U (2010) Wasserbilanzen der Region Berlin–Brandenburg. Diskussionspapier 7, Berlin-Brandenburgische Akademie der Wissenschaften, Interdisziplinäre Arbeitsgruppe »Globaler Wandel – Regionale Entwicklung«, Berlin, 57 pp. https://edoc.bbaw.de/files/278/diskussionspapier_7_gruenewald_online.pdf – last accessed in July 2022.

Gulev SK, Thorne PW, Ahn J, Dentener FJ, Domingues CM et al. (2021) Changing State of the Climate System. In: Masson-Delmotte V, Zhai P, Pirani A, Connors SL, Péan C et al. (eds) Climate Change 2021: The Physical Science Basis. Contribution of Working Group I to the Sixth Assessment Report of the Intergovernmental Panel on Climate Change. Cambridge University Press, Cambridge, UK, and New York, NY, USA, pp 287–422. doi:10.1017/9781009157896.004

Hoymann J, Dekkers J, Koomen E (2016) Szenarien zur Siedlungsflächenentwicklung. In: Wechsung F, Hartje V, Kaden S, Venohr M, Hansjürgens B, Gräfe P (eds) Die Elbe im globalen Wandel – Eine integrative Betrachtung. Vol. 9 in series Konzepte für die nachhaltige Entwicklung einer Flusslandschaft. Chapter 2.6, pp 135–175. Schweizerbart Science Publishers, Stuttgart, Germany. ISBN 978-3-510-65306-5.

Hübler M, Klepper G (2007) Kosten des Klimawandels – Die Wirkung steigender Temperaturen auf Gesundheit und Leistungsfähigkeit. Aktualisierte Fassung 07/2007. Institut für Weltwirtschaft, Kiel, 65 pp. https://www.ifw-kiel.de/fileadmin/Dateiverwaltung/IfW-Publications/Michael_Huebler/2kosten-des-klimawandels-die-wirkung-steigender-temperaturen-auf-gesundheit-und-leistungsfahigkeit/Kosten_des_Klimawandels_WWF_IfW.pdf – Last accessed in July 2022.

ILO (2019) Working on a warmer planet: The impact of heat stress on labour productuivity and decent work. International Labour Office, Geneva, 103 pp. ISBN 978-92-2-132967-1 (print), ISBN 978-92-2-132968-8 (web pdf). https://www.ilo.org/wcmsp5/groups/public/---dgreports/---dcomm/---publ/documents/publication/wcms_711919.pdf – Last accessed in July 2022.

Jendritzky G (1990) Methodik zur räumlichen Bewertung der thermischen Komponente im Bioklima des Menschen: fortgeschriebenes Klima-Michel-Modell. Veröffentlichungen der Akademie für Raumforschung und Landesplanung 114, Hannover, 80 pp. ISSN 0587-2642. ISBN 3-88838-207-6.

Jendritzky G, Sönning W, Swantes HJ (1979) Ein objektives Bewertungsverfahren zur Beschreibung des thermischen Milieus in der Stadt- und Landschaftsplanung („Klima-Michel-Modell“). Veröffentlichungen der Akademie für Raumforschung und Landesplanung 28, Hannover, 85 pp. ISSN 0587-2642. ISBN 3-507-91498-0.

Jendritzky G, Bröde P, Fiala D, Havenith G, Weihs P, Batchvarova E, DeDear R (2010) Der Thermische Klimaindex UTCI. DWD Klimastatusbericht 2009, pp 96–101. Deutscher Wetterdienst, Wiesbaden. https://www.dwd.de/DE/leistungen/klimastatusbericht/publikationen/ksb2009_pdf/artikel11.pdf – Last accessed in July 2022.

Koch H, Kaltofen M, Grünewald U, Messner F, Karkuschke M, Zwirner O, Schramm M (2005) Scenarios of water resources management in the Lower Lusatian mining district, Germany. Ecol Eng 24(1–2):49–57. doi:10.1016/j.ecoleng.2004.12.006

Kottek M, Grieser J, Beck C, Rudolf B, Rubel F (2006) World Map of the Köppen–Geiger climate classification updated. Meteorol Z 15(3):259–263. doi:10.1127/0941-2948/2006/0130

LAK Energiebilanzen (2022) Energiebilanzen. Website with interactive data tables. Länderarbeitskreis Energiebilanzen, Statistisches Landesamt Bremen. https://www.lak-energiebilanzen.de/ – last accessed in July 2022.

Lan L, Lian Z, Pan L, Ye Q (2009) Neurobehavioral approach for evaluation of office workers' productivity: The effects of room temperature. Build Environ 44(8):1578–1588. doi:10.1016/j.buildenv.2008.10.004

Maretzke S, Hoymann J, Schlömer C, Stelzer A (2021) Raumordnungsprognose 2040 – Bevölkerungsprognose: Ergebnisse und Methodik. BBSR-Analysen KOMPAKT 03/2021, 24 pp. Bundesinstitut für Bau-, Stadt- und Raumforschung im Bundesamt für Bauwesen und Raumordnung, Bonn, Germany. ISBN 978-3-87994-626-6. https://www.bbsr.bund.de/BBSR/DE/veroeffentlichungen/analysen-kompakt/2021/ak-03-2021-dl.pdf – Last accessed in January 2022.

MLUK (2021) Waldzustandsbericht 2021 des Landes Brandenburg. Ministerium für Landwirtschaft, Umwelt und Klimaschutz des Landes Brandenburg. Potsdam, 48 pp. https://mluk.brandenburg.de/sixcms/media.php/9/Waldzustandsbericht-2021.pdf – Last accessed in January 2022.

MULE (2021) Waldzustandsbericht Sachsen-Anhalt 2021. Ministerium für Umwelt, Landwirtschaft und Energie Sachsen-Anhalt (MULE), Magdeburg, 44 pp. https://www.nw-fva.de/fileadmin/nwfva/common/veroeffentlichen/wzb/WZB_SachsenAnhalt_2021.pdf – Last accessed in January 2022

Pohrt U, Kynast L, Templiner A (2021) Klimawandel und Bauunternehmen: Zunehmende Hitzebelastungen als Auswirkung des Klimawandels – Maßnahmen für Beschäftigte der Baubranche und ihre Gesundheit (Teil 2/4). BauPortal 2021(2):48–50. https://bauportal.bgbau.de/bauportal-22021/thema/branchenuebergreifend/serie-klimawandel-und-bauunternehmen-teil-2/4 – Last accessed in July 2022.

Porras-Salazar JA, Schiavon S, Wargocki P, Cheung T, Tham KW (2021) Meta-analysis of 35 studies examining the effect of indoor temperature on office work performance. Building and Environment 203:108037. doi:10.1016/j.buildenv.2021.108037

Port of Hamburg (2022) Seaborne Cargo Handling: Total Handling. Online table in bar graph (numbers shown on mouseover). Port of Hamburg, Hamburg. https://www.hafen-hamburg.de/en/statistics/seabornecargohandling/ – Last accessed in July 2022.

Scholz E, Totsche O (2022) Wasserwirtschaftlicher Jahresbericht der LMBV mbH – Zeitraum 01.01.–31.12.2021. Lausitzer und Mitteldeutsche Bergbau-Verwaltungsgesellschaft mbH, Senftenberg, 57+101 pp. https://www.lmbv.de/wp-content/uploads/2022/05/Wasserwirtschaftlicher-Jahresbericht-2021.pdf – Last accessed in July 2022.

Simon M, Bekele V, Kulasová B, Maul C, Oppermann R, Řehák P (2005) Die Elbe und ihr Einzugsgebiet – Ein geographisch-hydrologischer und wasserwirtschaftlicher Überblick. Internationale Kommission zum Schutz der Elbe, Magdeburg, 258 pp. https://www.ikse-mkol.org/fileadmin/media/user_upload/D/06_Publikationen/07_Verschiedenes/2005_IKSE-Elbe-und-ihr-Einzugsgebiet.pdf – Last accessed in July 2022.

Simon S, Schöpfer R, Schumacher D, Meyer C (2019) Auswirkungen der Sommertrockenheit 2018 auf die öffentliche Wasserversorgung. Energie Wasser-Praxis 2019(3):14–19. https://www.dvgw.de/medien/dvgw/wasser/klimawandel/sommertrockenheit-wasserversorgung-energie-wasser-praxis-maerz-2019.pdf – Last accessed in July 2022.

SMEKUL (2021) Waldzustandsbericht 2021. Sächsisches Staatsministerium für Energie, Klimaschutz, Umwelt und Landwirtschaft (SMEKUL), Dresden, 64 pp. https://publikationen.sachsen.de/bdb/artikel/39012 – Last accessed in January 2022.

Staiger H, Bucher K, Jendritzky G (1997) Gefühlte Temperatur. Die physiologisch gerechte Bewertung von Wärmebelastung und Kältestress beim Aufenthalt im Freien in der Maßzahl Grad Celsius. Annalen der Meteorologie 33, Deutscher Wetterdienst, Offenbach, pp 100-107. https://www.dwd.de/DE/leistungen/pbfb_verlag_annalen/pdf_einzelbaende/33_pdf

Statistik BB (2017–2019) Gäste, Übernachtungen und Beherbergungskapazität. Statistischer Bericht G IV 1 - m, December Issues of the years 2016–2018 for Berlin and for Brandenburg. Statistik Berlin Brandenburg, Potsdam. https://www.statistik-berlin-brandenburg.de/archiv/g-iv-1-m – Last accessed in July 2022.

Statistik BB (2016–2020) Erzeugung in Aquakulturbetrieben im Land Brandenburg. Statistischer Bericht C III 11 – j / 20. Annual issues. Statistik Berlin Brandenburg, Potsdam. https://www.statistik-berlin-brandenburg.de/c-iii-11-j – Last accessed in July 2022.

Statistik ST (2019) Tourismus, Gastgewerbe: Gäste und Übernachtungen im Reiseverkehr, Beherbergungskapazität. Statistischer Bericht G IV m12/18, Dezember 2018. Sachsen-Anhalt, Statistisches Landesamt, Magdeburg, 51+25 pp. https://www.statistischebibliothek.de/mir/servlets/MCRFileNodeServlet/STHeft_derivate_00003489/6G401_12_18-A.pdf – Last accessed in July 2022.

Statistische Ämter (2014) Zensus 2011 – Bevölkerung nach Geschlecht, Alter, Staatsangehörigkeit, Familienstand und Religionszugehörigkeit – Endgültige Ergebnisse. Statistische Ämter des Bundes

und der Länder, Statistisches Landesamt Rheinland-Pfalz, Bad Ems, 69 pp.
https://www.zensus2011.de/SharedDocs/Downloads/DE/Publikationen/Aufsaetze_Archiv/2014_10_RP_Bevoelkerung.pdf – Last accessed in July 2022.

Statistische Ämter (2022) Bruttoinlandsprodukt, Bruttowertschöpfung in den Ländern der Bundesrepublik Deutschland 1991 bis 2021, Berechnungsstand: November 2021/Februar 2022. Excel file. Arbeitskreis »Volkswirtschaftliche Gesamtrechnungen der Länder«, Statistische Ämter des Bundes und der Länder.
http://www.statistikportal.de/de/veroeffentlichungen/bruttoinlandsprodukt-bruttowertschoepfung – Last accessed in July 2022.

SVUVK (2021) Waldzustandsbericht 2021 des Landes Berlin. Senatsverwaltung Umwelt, Verkehr und Klimaschutz (SVUVK); Berliner Forsten, Berlin. 27 pp.
https://www.berlin.de/forsten/_assets/waldschutz/waldzustandsberichte/waldzustandsbericht_2021.pdf – Last accessed in January 2022.

TMIL (2021) Waldzustandsbericht 2021 – Forstliches Umweltmonitoring in Thüringen. Thüringer Ministerium für Infrastruktur und Landwirtschaft (TMIL), Erfurt, 53 pp. https://infrastruktur-landwirtschaft.thueringen.de/fileadmin/Forst_und_Jagd/Forstwirtschaft/TMIL_Broschuere_Waldzustandsbericht_2021_11-21_Web.pdf – Last accessed in January 2022

Vicente-Serrano SM, López-Moreno JI, Beguería S, Lorenzo-Lacruz J, Azorin-Molina C, Morán-Tejeda E (2012) Accurate Computation of a Streamflow Drought Index. J Hydrol Eng 17(2):318–332. doi:10.1061/(ASCE)HE.1943-5584.0000433

Wellbrock N, Eickenscheidt N, Hilbrig L, Dühnelt P, Holzhausen M et al. (2018) Leitfaden und Dokumentation zur Waldzustandserhebung in Deutschland. Thünen Working Paper 84. Thünen-Institut, Eberswalde und Braunschweig, 97 pp.
https://literatur.thuenen.de/digbib_extern/dn059504.pdf – Last accessed in January 2022.

Wild M (2014) Global Dimming and Brightening. In: Freedman B (ed.) Global Environmental Change. Handbook of Global Environmental Pollution, vol 1, pp 39–47. Springer, Dordrecht. doi:10.1007/978-94-007-5784-4_2739-47

Wild M, Wacker S, Yang S, Sanchez-Lorenzo A (2021) Evidence for Clear-Sky Dimming and Brightening in Central Europe. Geophys Res Lett 48(6):e2020GL092216. doi:10.1029/2020GL092216

Zhao M, Lee JKW, Kjellstrom T, Cai W (2021) Assessment of the economic impact of heat-related labor productivity loss: a systematic review. Climatic Change 167(1–2):22 doi:10.1007/s10584-021-03160-7
